# A machine learning-derived immune-related prognostic model identifies *PLXNA3* as a functional risk gene in colorectal cancer

**DOI:** 10.3389/fimmu.2025.1653794

**Published:** 2025-09-02

**Authors:** Hanzhang Lyu, Song Wang, Guodong Guo, Weiteng Lin, Chenshen Huang, Hong Chen, Chao Xu, Liming Liu, Qi Huang, Fangqin Xue

**Affiliations:** ^1^ Shengli Clinical Medical College of Fujian Medical University, Fujian Provincial Hospital, Fuzhou, China; ^2^ Fuzhou University Affiliated Provincial Hospital, Fuzhou University School of Medicine, Fuzhou, China; ^3^ Department of General Surgery, Tongji Hospital, School of Medicine, Tongji University, Shanghai, China; ^4^ Department of General Surgery, Shibei Hospital of Shanghai Jingan District, Shanghai, China

**Keywords:** colorectal cancer, *PLXNA3*, prognostic model, immune microenvironment, machine learning, single-cell RNA sequencing, spatial transcriptomics, drug sensitivity

## Abstract

**Introduction:**

Colorectal Cancer (CRC) remains a leading cause of cancer-related mortality, characterized by substantial interpatient heterogeneity and limited effective prognostic biomarkers.

**Methods:**

To address this gap, we constructed a robust prognostic model by integrating over 100 machine learning algorithms—such as LASSO, CoxBoost, and StepCox—based on transcriptomic and clinical data from The Cancer Genome Atlas (TCGA) and Gene Expression Omnibus (GEO) cohorts.

**Results:**

Plexin-A3 (*PLXNA3*) emerged as a top risk gene within the ensemble model, which achieved strong predictive performance, surpassing conventional clinical indicators. Multi-omics validation confirmed *PLXNA3’s* prognostic relevance. Spatial and single-cell transcriptomics demonstrated their enrichment in malignant epithelial regions and negative association with immune cell infiltration, particularly CD8^+^ T cells and plasma cells. Transcription factor (TF) and microRNA (miRNA) correlation analyses revealed potential upstream regulators of *PLXNA3* linked to tumor stemness and immune suppression. Functional enrichment indicated its association with cell cycle, DNA damage repair, and interferon signaling pathways. Immunohistochemistry (IHC) confirmed *PLXNA3* overexpression in tumor tissues and its correlation with nodal metastasis. Moreover, drug sensitivity profiling and Connectivity Map (CMap) analysis identified potential compounds, including imatinib, MS-275 and fasudil, capable of reversing *PLXNA3*-driven transcriptional programs.

**Discussion:**

This study identifies *PLXNA3* as a novel immune-related biomarker in colorectal cancer and elucidates its multifaceted role in tumor progression, immune evasion, and therapeutic resistance. These findings provide a foundation for incorporating *PLXNA3* into precision oncology frameworks for gastrointestinal malignancies.

## 1 Introduction

CRC is among the most common and lethal malignancies worldwide, ranking third in incidence and second in cancer-related mortality (1). Despite progress in early detection and therapeutic advances, the prognosis of CRC patients remains highly variable (2). This heterogeneity is largely attributed to the diverse genetic landscape and complex tumor microenvironment (TME), which are not fully captured by conventional clinicopathological staging (3).

Recent advances in transcriptomics and bioinformatics have enabled more precise prognostic modeling, particularly through the integration of high-throughput expression data with machine learning frameworks (4). Traditional statistical methods such as logistic regression, have been extensively applied to identify prognostic biomarkers in cancer. However, their performance is often limited when dealing with high-dimensional, non-linear, and heterogeneous biological datasets (5). In contrast, machine learning (ML) and ensemble learning models—particularly those employing stacked or hybrid architectures—have shown superior performance in capturing complex patterns in omics data (6, 7). In recent years, multi-model integration strategies have emerged as a dominant trend, offering improved generalizability and robustness in prognostic prediction (8).

Here, our study employed over 100 machine learning algorithms in a stacked ensemble framework to construct a novel immune-related prognostic model for CRC. Among the candidate genes identified, we focused on *PLXNA3*, a member of the plexin family (9), due to its consistent high-risk signal across models. Although *PLXNA3* has been implicated in neural development and tumor metastasis in several malignancies (10–12), its role in gastrointestinal cancers remains largely uncharacterized.

To explore the prognostic relevance and biological function of *PLXNA3*, we initiated our investigation with a pan-cancer screening strategy to broadly evaluate its expression patterns and clinical significance across multiple tumor types. This led to the observation of elevated *PLXNA3* expression in gastrointestinal malignancies, particularly in colorectal, gastric, and esophageal cancers. Building on this, we conducted comparative analyses within gastrointestinal cancers to highlight the specific prognostic and immunological role of *PLXNA3* in CRC.

**Figure 1:**
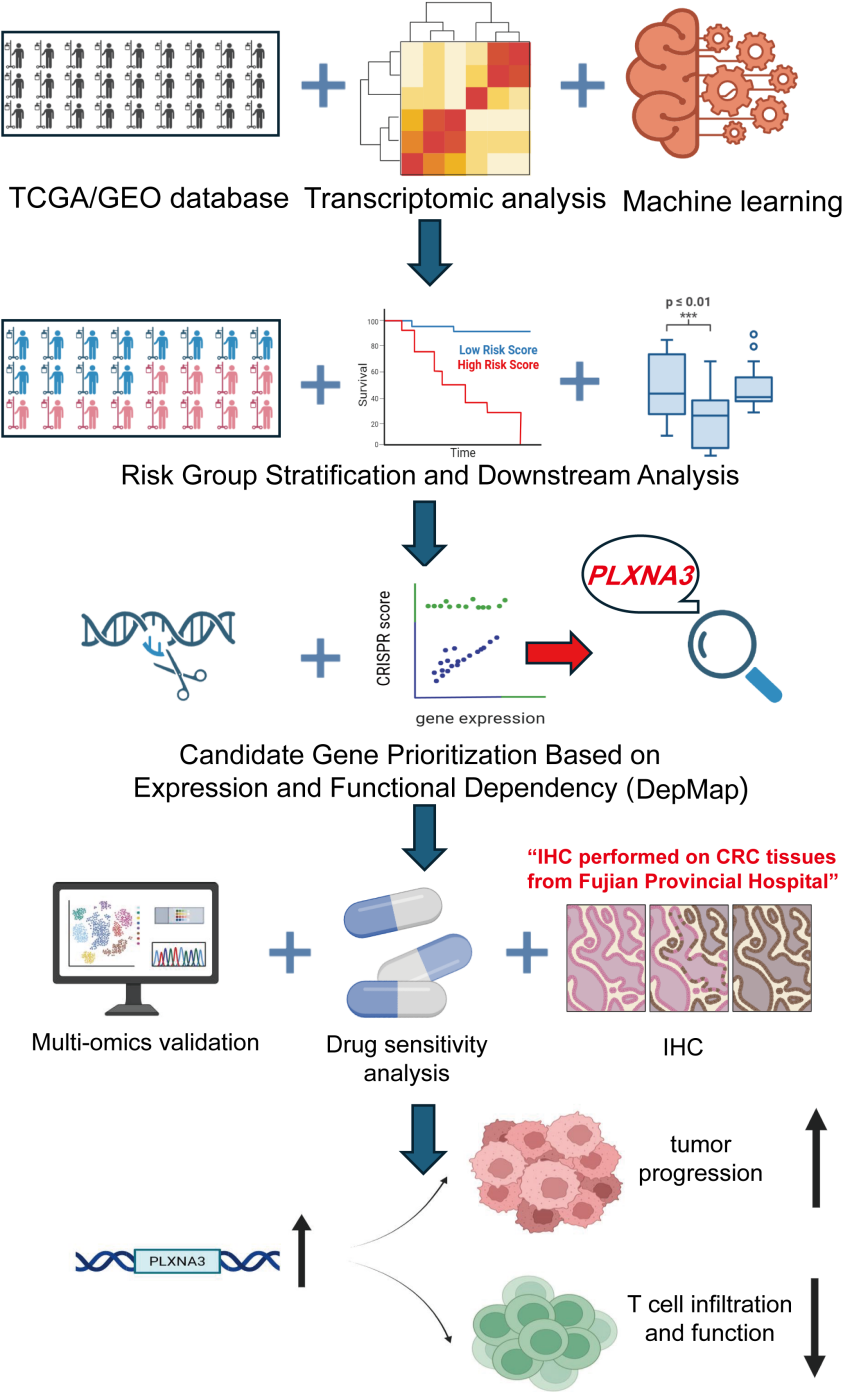
Overview of the study design and analytical workflow. Transcriptomic and clinical data from TCGA and GEO cohorts were integrated and processed using over 100 machine learning algorithms to construct an immune-related prognostic model for colorectal cancer. *PLXNA3* was identified as a key risk gene based on risk group stratification and functional dependency analysis using DepMap. Subsequent validation included spatial transcriptomics, single-cell RNA sequencing, transcriptional and post-transcriptional regulatory profiling, immunohistochemistry (IHC), and drug sensitivity analysis. The study further explored the biological role of *PLXNA3* in tumor progression and T cell immune exclusion.

We subsequently focused our study on CRC, integrating bulk transcriptomic, spatial transcriptomic, single-cell RNA sequencing, immunohistochemistry, and pharmacogenomic data to comprehensively assess *PLXNA3* at multiple biological levels. An overall workflow illustrates the design of the study, from initial screening and model construction to CRC-specific multi-omics validation (**Figure 1**). Through this multilayered approach, we aimed to delineate the spatial, cellular, and functional characteristics of *PLXNA3*, and to assess its potential as a prognostic biomarker and therapeutic target in CRC.

## 2 Methods and materials

### 2.1 Data collection and preprocessing

Transcriptomic and clinical data for Colon Adenocarcinoma (COAD) were obtained from TCGA through the Genomic Data Commons (https://portal.gdc.cancer.gov/). External validation data were retrieved from the GEO dataset GSE39582 (13). Normal tissue expression data were sourced from the Genotype-Tissue Expression (GTEx) project (14). Transcript-per-million (TPM) values from TCGA and GTEx were merged and standardized using Z-score transformation to enable cross-cohort comparability. Immune-related gene lists were acquired from the Immunology Database and Analysis Portal (ImmPort, https://www.immport.org/. Gene dependency scores, including CRISPR-based essentiality metrics, were downloaded from the DepMap portal (https://depmap.org/portal/). Protein-level expression data for *PLXNA3* were obtained from the Human Protein Atlas (HPA), and integrated tissue-level TPM values were accessed via the UCSC Xena platform.

### 2.2 Differential expression and prognostic analysis

Differentially expressed immune-related genes between tumor and adjacent normal tissues were identified using the `DESeq2` package in R (15), with cutoff thresholds set at |log2 fold change| > 1.5 and false discovery rate (FDR) < 0.05. Prognostic evaluation was performed using univariate Cox proportional hazards regression and Kaplan–Meier (KM) survival analysis. The `survival` (16) and `survminer` (17) packages were used to compute hazard ratios (HR) and generate KM curves. High and low expression groups were defined using the optimal cutoff determined by `surv_cutpoint` (17) while enforcing a minimum group proportion of 30% to avoid over-segmentation. Log-rank tests were applied to evaluate statistical significance between survival curves. A summary heatmap was constructed to visualize significance patterns across cancer types and survival endpoints including overall survival (OS), disease-specific survival (DSS), progression-free interval (PFI), and disease-free interval (DFI).

### 2.3 Machine learning model construction

To construct a robust prognostic model, we implemented a stacked ensemble learning framework that integrated over 100 machine learning algorithms. Candidate models included LASSO regression, CoxBoost, elastic net, and plsRcox. Feature selection was performed using recursive elimination and cross-validation. Model performance was evaluated using the concordance index (C-index), and the model with the highest C-index was selected for downstream validation. The training cohort was derived from TCGA-COAD, and external validation was conducted using the GSE39582 dataset. Time-dependent ROC (Receiver operating characteristic) curves and C-index curves were generated to compare predictive accuracy with conventional clinical indicators. For both the TCGA (training) and GSE39582 (validation) cohorts, risk scores for each patient were calculated using the final selected ensemble prognostic model (CoxBoost + SuperPC). Patients were then stratified into high-risk and low-risk groups based on the median value of the risk score within each respective cohort. This median-based dichotomization enabled consistent and data-specific stratification, facilitating survival comparison and model validation across datasets.

### 2.4 CRISPR-based functional dependency analysis using DepMap

Gene dependency scores were obtained from the DepMap Public 22Q2 release via the DepMap portal (https://depmap.org/portal/) . The CERES-corrected CRISPR knockout scores, which estimate the effect of gene inhibition on cell viability, were used to quantify gene essentiality across a broad panel of cancer cell lines (18). *PLXNA3* and other candidate genes were evaluated for their dependency scores in colorectal cancer cell lines. Data processing and integration with gene expression profiles were conducted in Python using the Pandas and Seaborn packages.

### 2.5 Pan-cancer expression and diagnostic efficacy

*PLXNA3* expression profiles across 33 cancer types were analyzed using merged TCGA and GTEx datasets. TPM values were standardized via Z-score transformation for cross-tumor comparison. ROC curves were generated using the `pROC` package (19) to assess diagnostic accuracy, and area-under-the-curve (AUC) values were reported separately for TCGA-only and TCGA-GTEx merged datasets.

### 2.6 Spatial transcriptomic analysis

Spatial transcriptomic data from ten colorectal cancer tissue sections were obtained from the Sparkle database (https://grswsci.top/). The Sparkle database, following previous studies (20, 21), integrates 10x Visium spatial transcriptomic data to construct a pan-cancer spatial atlas. By characterizing the cell types within each spot, regions were annotated according to the predominant cell type based on proportional composition. Raw count matrices were normalized using Seurat’s `NormalizeData` function (22). Gene expression was visualized using `SpatialFeaturePlot` (22). Tissue spots were categorized into malignant (Mal) and non-malignant (nMal) regions based on prior deconvolution analysis, and Wilcoxon tests were used to compare *PLXNA3* expressions between zones. Expression heatmaps were generated across dominant cell-type zones using Z-score–scaled values.

### 2.7 Single-cell transcriptomic analysis

Single-cell RNA sequencing (ScRNA-seq) data from gastrointestinal tumors were analyzed via the Tumor Immune Single-cell Hub2 (TISCH2) portal (23). Cell-type–specific expression levels of *PLXNA3* were visualized with heatmaps and bar plots. Z-score transformation was conducted to evaluate associations between *PLXNA3* expression and immune cell proportions (e.g., pDCs, CD8^+^ T cells). Correlation results were visualized with lollipop plots and scatter curves. Multi-method immune infiltration correlations were generated and plotted using `ComplexHeatmap` (24).

### 2.8 Transcription factor correlation analysis

Expression data for *PLXNA3* and transcription factors were obtained from the normalized RNA-seq dataset EBPlusPlusAdjustPANCAN_IlluminaHiSeq_RNASeqV2.geneExp.tsv (25), available through the TCGA Pan-Cancer Atlas project (https://gdc.cancer.gov/about-data/publications/pancanatlas) . Log2 transformation was applied to gene expression values for normalization. Pearson correlation coefficients between *PLXNA3* and all transcription factors were calculated using the cor.test function (method = “Pearson”) in R (version 4.3.3). For stratification analysis, samples were divided into four quartiles (Q1–Q4) based on transcription factor expression levels. Differential expression of *PLXNA3* across quartiles was evaluated using the Kruskal–Wallis test (kruskal.test function), enabling detection of expression trends across TF-defined subgroups. For miRNA association analysis, predicted *PLXNA3*-related miRNAs were obtained from the grswsci platform (https://grswsci.top/) . Pearson correlations were computed using cor.test, and statistical enrichment was assessed using Fisher’s exact test.

### 2.9 Functional and immunological pathway analysis

Functional correlations between *PLXNA3* and cellular states (e.g., proliferation, cell cycle, DNA repair) were evaluated using CancerSEA (26) across pan-gastrointestinal tumors. gene set enrichment analysis (GSEA) was performed with the `clusterProfiler` package against Hallmark and Kyoto Encyclopedia of Genes and Genomes (KEGG) gene sets (27). Enrichment results were stratified by *PLXNA3* expression levels. Pairwise Pearson correlations between *PLXNA3* and immune-related genes in gastrointestinal tumors were computed using `cor.test` and presented as complex heatmaps.

### 2.10 Immunohistochemistry staining and scoring

Twenty-two colorectal cancer samples were collected for immunohistochemical validation. All specimens were obtained from postoperative colorectal cancer patients at Fuzhou University Affiliated Provincial Hospital, and the sample acquisition was approved by the hospital’s Ethics Committee (Approval number: K2024-12-064). Among them, ten paired tumors and adjacent normal tissues were used for quantitative analysis. Formalin-fixed paraffin-embedded sections were stained with anti-*PLXNA3* antibodies. Representative images were acquired at 5×, 20× magnifications. Staining intensity was graded on a 0–3 scale, and IHC scores were calculated as intensity × positive cell percentage (28). Group comparisons were made using two-tailed Student’s t-tests in GraphPad Prism. Correlations between IHC score and Tumor–Node–Metastasis (TNM) staging were visualized using scatter plots.

### 2.11 Drug sensitivity and CMap analysis

Drug response profiles were downloaded from Profiling Relative Inhibition Simultaneously in Mixtures (PRISM), Cancer Therapeutics Response Portal (CTRP), and Genomics of Drug Sensitivity in Cancer (GDSC) datasets (29–31). Pearson correlation coefficients were calculated between *PLXNA3* expression and AUC drug sensitivity metrics. Strongly associated compounds in GDSC1 and GDSC2 were prioritized. CMap analysis was performed using the eXtreme Sum (XSum) method to identify candidate small molecules predicted to reverse the transcriptional signature associated with *PLXNA3* overexpression. Among the 1288 compounds screened, those with the lowest connectivity scores were highlighted as potential therapeutic inhibitors (32).

## 3 Result

### 3.1 Immune-related gene expression and prognostic model construction

**Figure 2:**
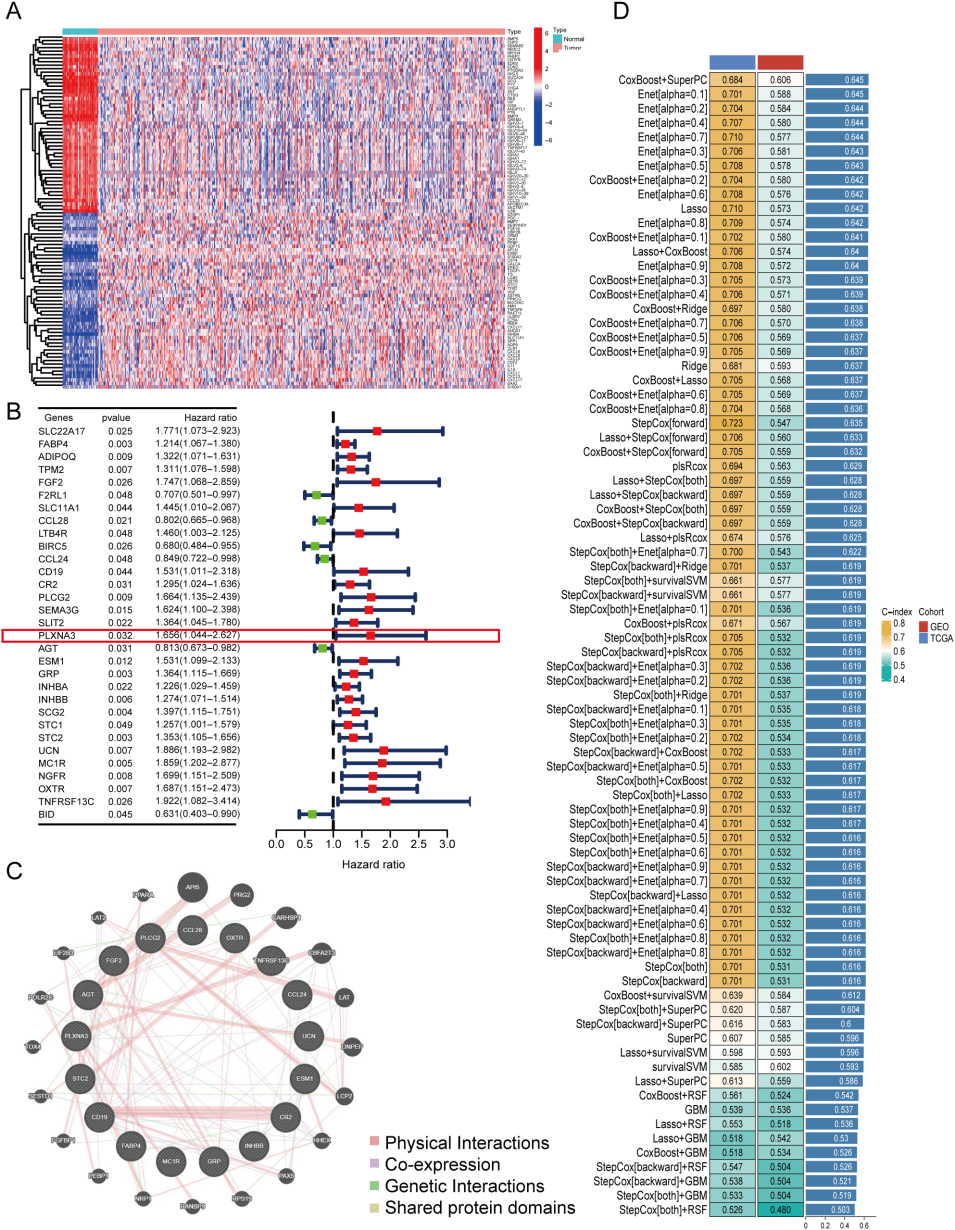
Identification of immune-related prognostic genes and construction of a multi-model prognostic risk model. **(A)** Heatmap of differentially expressed immune-related genes between tumor and adjacent normal tissues in TCGA-COAD (log2 fold change > 1.5, FDR < 0.05). **(B)** Forest plot of univariate Cox regression analysis showing hazard ratios (HR) for selected immune-related genes associated with overall survival in TCGA-COAD. *PLXNA3* was identified as a significant high-risk gene (HR = 1.656, p = 0.032). **(C)** Protein-protein interaction (PPI) network of prognostic immune-related genes constructed using the GeneMANIA database. **(D)** Performance comparison of ensemble machine learning models using integrated TCGA and GEO datasets. The selected prognostic model achieved the highest concordance index (C-index = 0.645) and was used for downstream survival analysis.

To identify immune-related prognostic biomarkers in COAD, we first integrated transcriptomic profiles from 461 COAD samples in TCGA with immune gene sets curated from the Immunology Database and Analysis Portal (ImmPort). This yielded a comprehensive dataset of immune-related gene expressions specific to COAD. Differential expression analysis between tumor and adjacent normal tissues revealed substantial immune heterogeneity, as visualized by a heatmap highlighting significantly dysregulated genes (**Figure 2A**).

Next, we performed univariate Cox regression analysis by combining the expression profiles of differentially expressed immune genes with matched clinical outcomes from the TCGA cohort. This analysis identified a set of immune genes significantly associated with OS. Among these, *PLXNA3* emerged as one of the top high-risk genes (HR=1.656, 95% CI: 1.004–2.267, p=0.032), suggesting its potential role in driving adverse clinical outcomes (**Figure 2B**).

To investigate potential biological interactions among the survival-related immune genes, a protein-protein interaction (PPI) network was constructed using the GeneMANIA platform. The resulting network (**Figure 2C**) illustrated functional connectivity among these genes, implying their involvement in immune regulation and tumor signaling.

Building on these findings, we developed a robust prognostic model by integrating immune-related gene expression data from both TCGA and an independent GEO cohort (GSE39582). Using a stacked ensemble machine learning framework incorporating over 100 algorithms—including LASSO, Coxboost, and StepCox—we identified the model with the highest concordance index (C-index = 0.645) as our final prognostic model (**Figure 2D**). This model demonstrated consistent accuracy in both training (TCGA, C-index = 0.684) and validation (GEO, C-index = 0.606) datasets, establishing a reliable foundation for COAD prognosis prediction.

Notably, the top-performing model—based on the integration of CoxBoost and SuperPC—yielded 17 high-weighted genes, including well-established immune regulators (e.g., *CD19, CCL24, CCL28, PLCG2* and *FABP4*) *(* 33–37). Among these, *PLXNA3* emerged as a gene of particular interest due to its comparable model-derived importance score, coupled with its limited prior characterization in colorectal cancer immunity. The full list of model-scoring genes and their hazard ratios is provided in **Supplementary Table S2**.

### 3.2 Survival analysis and validation of the prognostic model

**Figure 3:**
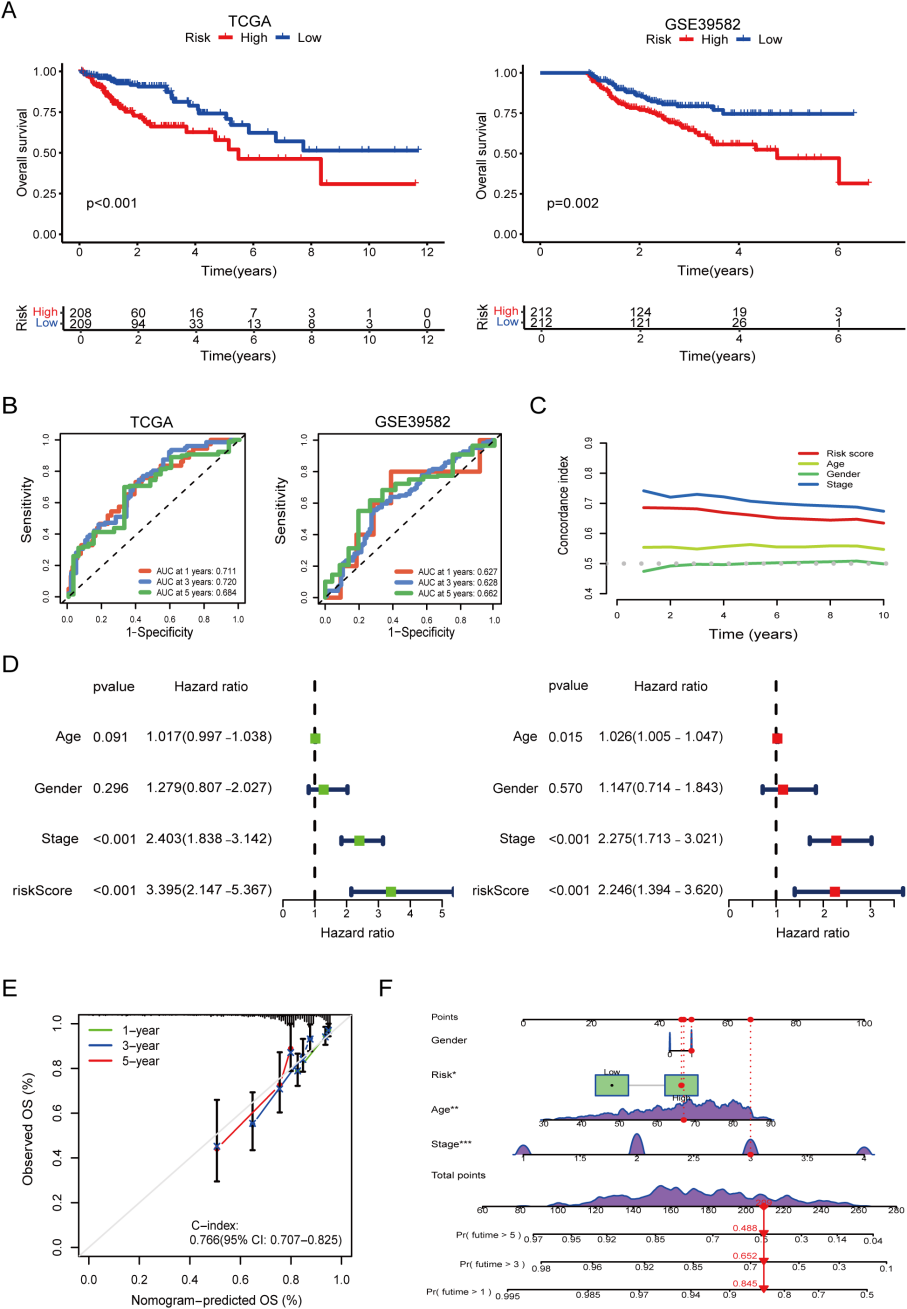
Survival validation and clinical utility of the prognostic model, risk groups were stratified using surv_cutpoint. **(A)** Kaplan–Meier survival curves demonstrating significantly poorer overall survival in high-risk groups in both TCGA and GEO validation cohorts (log-rank p < 0.001). **(B)** Time-dependent receiver operating characteristic (ROC) curves showing high predictive performance of the model in TCGA and GEO. **(C)** Comparison of model performance with clinical features using time-dependent concordance index, indicating superior prognostic value of the model across follow-up years. **(D)** Univariate and multivariate Cox regression analysis showing that the model-derived risk score is an independent predictor of overall survival (multivariate HR = 2.246, p < 0.001). **(E)** Calibration curves confirming the agreement between predicted and actual survival probabilities. **(F)** Nomogram combining the risk score with clinical variables for individualized survival prediction. The asterisks (*, **, ***) next to variables in the nomogram indicate statistical significance derived from the Cox regression analysis used for model construction, consistent with conventional notation (*p < 0.05, **p < 0.01, ***p < 0.001).

To evaluate the clinical utility of our prognostic model, we performed KM survival analyses using both the TCGA and GEO (GSE39582) datasets. As shown in **Figure 3A**, patients stratified into the high-risk group had significantly worse OS than those in the low-risk group in both cohorts (log-rank test, p < 0.001 in TCGA; p = 0.002 in GSE39582), underscoring the model’s robust discriminative ability across independent populations.

ROC curve analysis further confirmed the model’s predictive accuracy at different time points. In the TCGA cohort, the AUC reached 0.711, 0.720, and 0.684 for 1-, 3-, and 5-year survival, respectively, indicating strong temporal consistency (**Figure 3B**). In the GEO cohort, the AUCs were 0.627, 0.628, and 0.662, suggesting moderate external generalizability.

To assess the added prognostic value beyond standard clinical indicators, we compared our model’s predictive performance against common clinicopathological features. Combined ROC analyses demonstrated that the risk score achieved competitive or superior predictive ability, and time-dependent C-index curves revealed consistent long-term prognostic accuracy (**Figure 3C**).

Univariate and multivariate Cox regression analyses based on TCGA data confirmed that the model-derived risk score served as an independent prognostic factor. In the multivariate analysis, the risk score yielded a HR of 2.2446 (95% CI: 1.394–3.620, p < 0.001), second only to TNM stage (HR = 2.275, 95% CI: 1.713–3.021, p < 0.001). Notably, in the univariate setting, the risk score demonstrated even stronger predictive power than TNM stage (**Figure 3D**).

Finally, calibration analysis showed excellent agreement between predicted and observed survival at 1, 3, and 5 years. The nomogram integrating the risk score with clinical parameters yielded a C-index of 0.766 (95% CI: 0.707–0.825), supporting its potential for individualized prognostic assessment in clinical practice (**Figures 3E, F**).

### 3.3 Comparison of immune functional differences between high- and low-risk groups

To explore the immunological landscape associated with our prognostic model, we stratified TCGA-COAD samples into high- and low-risk groups and analyzed immune-related characteristics across multiple dimensions.

**Figure 4:**
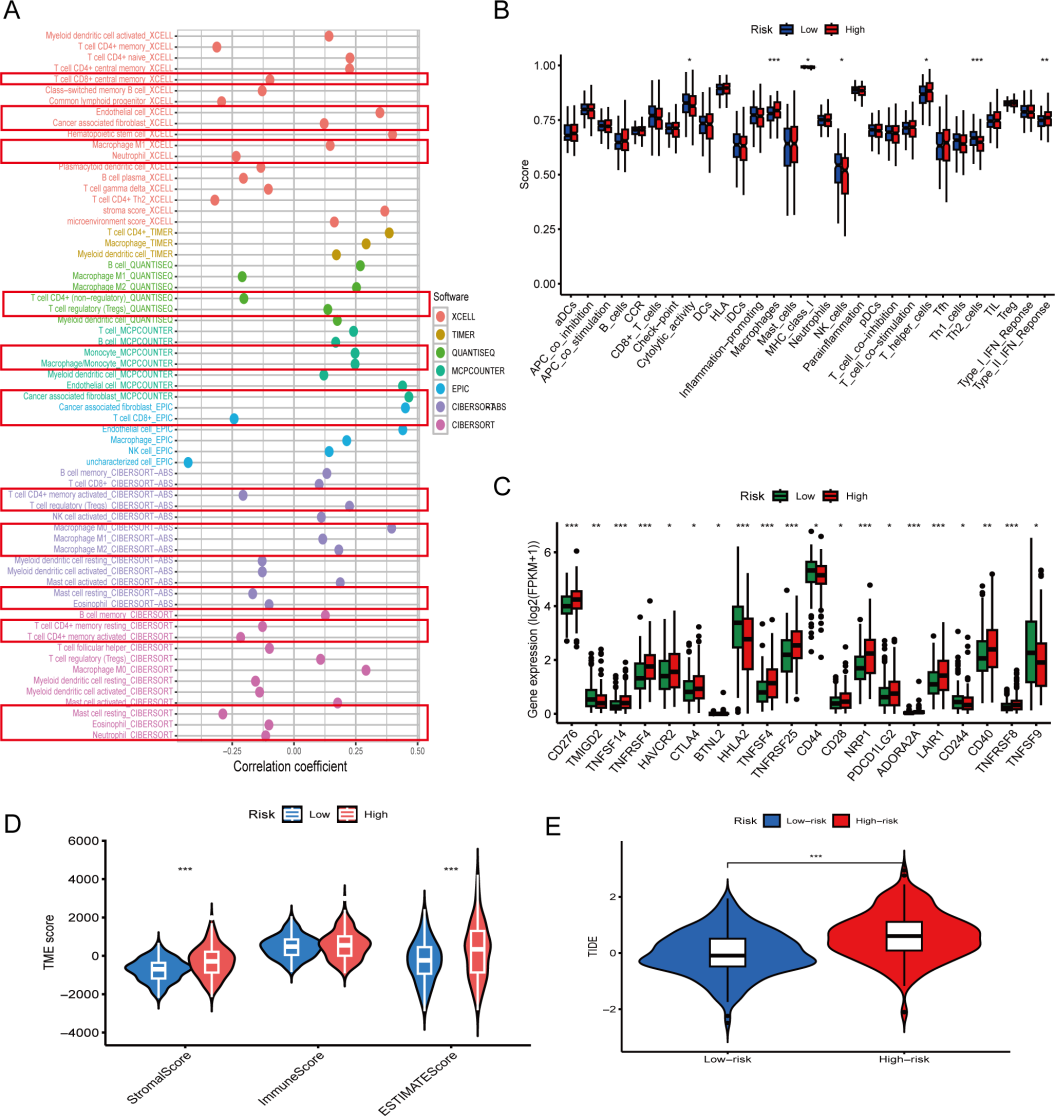
Immune microenvironment characteristics associated with the prognostic model. **(A)** Correlation between risk scores and immune cell infiltration levels using multiple computational algorithms (e.g. XCell, TIMER, MCPcounter). CD8^+^ T cells and CD4^+^ T cells show negative correlations, while Tregs and cancer associated fibroblast show positive correlations. **(B)** Boxplots comparing immune cell infiltration scores between high- and low-risk groups. Immune effector cells such as Th2 cells are significantly reduced in high-risk patients (p < 0.01). **(C)** Expression of immune checkpoint genes including *CD276* and *CTLA4* is significantly higher in the high-risk group (p < 0.05). **(D)** Tumor microenvironment (TME) scores: high-risk patients exhibit lower immune scores and higher stromal scores (p < 0.01). **(E)** Tumor Immune Dysfunction and Exclusion (TIDE) scores are significantly elevated in high-risk patients, indicating potential immune escape (p < 0.001). The asterisks (*, **, ***) in the figures indicate statistical significance levels: p < 0.05 (*), p < 0.01 (**), p < 0.001 (***).

First, correlation analysis between immune cell infiltration levels and risk scores revealed distinct patterns (**Figure 4A**). Key effector cells—including CD8^+^ T cells, CD4^+^ T cells, Mast cells, eosinophils and neutrophils—showed significant negative correlations with risk scores, while immunosuppressive populations such as regulatory T cells (Tregs), tumor-associated fibroblasts and macrophages were positively correlated. These findings suggest that high-risk tumors are characterized by reduced immune activation and enhanced immunosuppressive cell infiltration.

Consistently, comparisons of immune cell abundance between risk groups confirmed that low-risk patients exhibited significantly higher Cytolytic activity and higher infiltration of NK cells, and Th2 cells, whereas high-risk patients demonstrated enrichment of macrophages and IFN response (**Figure 4B**, p < 0.01). These immune infiltration profiles indicate an immunosuppressive tumor microenvironment in high-risk COAD.

Immune subtyping analysis showed no statistically significant distribution differences between groups (p = 0.13; **Supplementary Figure 1**) and was therefore not considered further.

We investigated the expression of immune checkpoint-related genes between high- and low-risk groups. Several inhibitory markers, including *CD276*, *HAVCR2*, *CTLA4*, *PDCD1LG2*, *LAIR1*, and *ADORA2A*, were significantly upregulated in the high-risk group (**Figure 4C**, p < 0.05), suggesting an immunosuppressive tumor microenvironment.

Further, TME scoring revealed that high-risk patients had significantly lower immune scores and higher stromal scores (**Figure 4D**), consistent with a less inflamed, stromal-dominant phenotype.

Finally, TIDE (Tumor Immune Dysfunction and Exclusion) analysis demonstrated significantly elevated immune escape scores in the high-risk group (p < 0.001; **Figure 4E**), reinforcing the concept that these tumors may evade immune surveillance more effectively and show reduced responsiveness to immunotherapy.

### 3.4 Prognostic significance and pan-cancer expression analysis of *PLXNA3*

**Figure 5:**
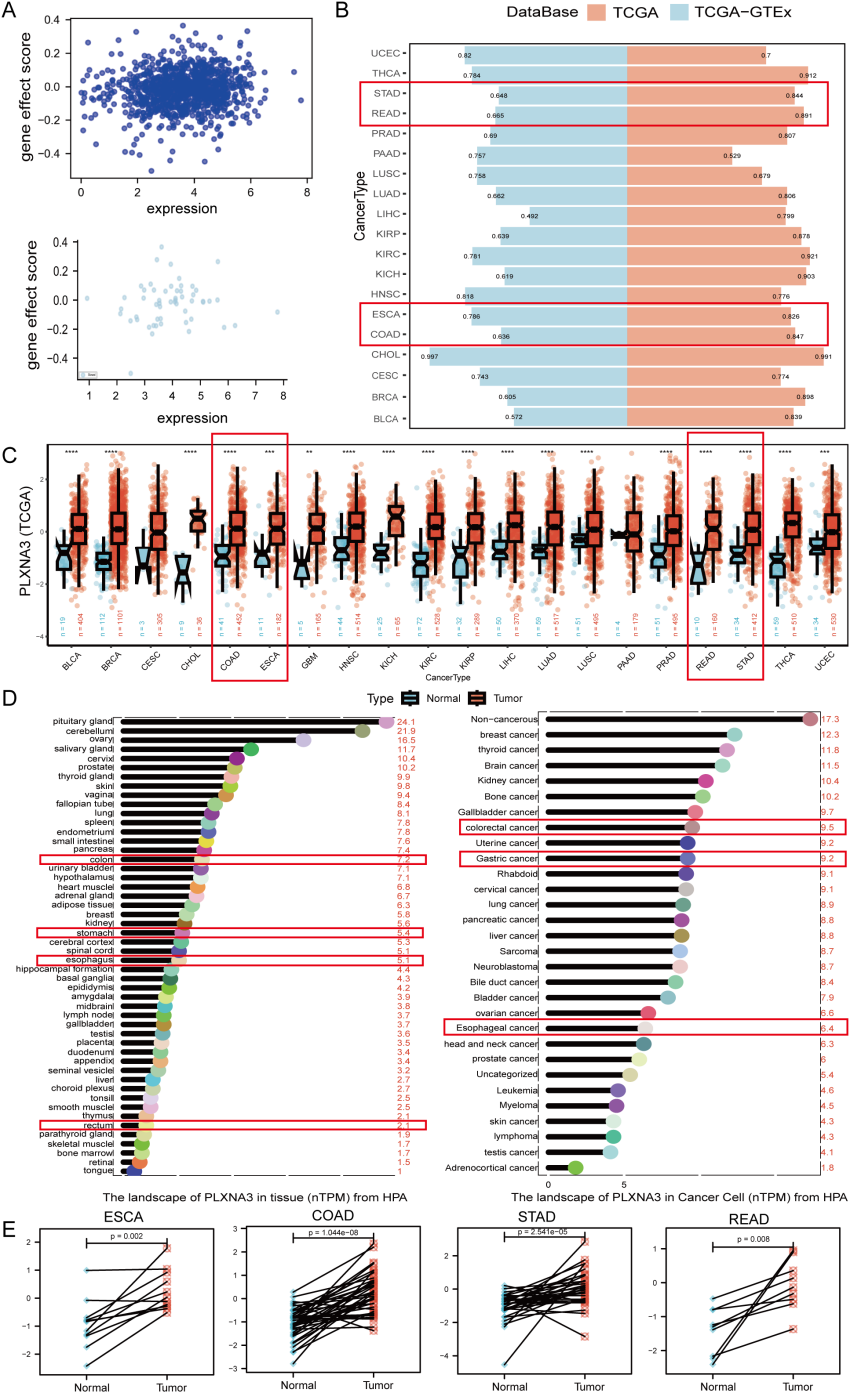
Expression pattern and diagnostic potential of *PLXNA3* in pan-gastrointestinal cancers. **(A)** Correlation between *PLXNA3* expression and gene effect scores from CRISPR data. **(B)** AUC values of *PLXNA3* in tumor–normal classification across TCGA and TCGA-GTEx cohorts. **(C)** *PLXNA3* expression in tumor vs. normal tissues across TCGA cancer types. **(D)** *PLXNA3* transcript levels in normal tissues and cancer cell types (HPA data). **(E)** Paired expression comparison in COAD, READ, STAD, and ESCA (Wilcoxon test). The asterisks (*, **, ***) in the figures indicate statistical significance levels: p < 0.05 (*), p < 0.01 (**), p < 0.001 (***).

Following the identification of 17 prognostic immune-related genes through our machine learning pipeline (**Figure 2D**), we performed cross-platform prioritization using gene expression profiles and CRISPR dependency scores from the DepMap database. *PLXNA3* stood out for its combination of high expression and strong dependency across multiple digestive system cancer cell lines, highlighting its potential functional relevance in tumor maintenance and positioning it as a candidate of interest for downstream investigation (**Figure 5A**).

To assess the diagnostic performance of *PLXNA3*, we conducted ROC curve analyses across tumor types using the pROC package. In the TCGA cohort, *PLXNA3* demonstrated excellent discriminatory power in gastrointestinal malignancies, including Esophageal Carcinoma (ESCA), Stomach Adenocarcinoma (STAD), COAD, and Rectum Adenocarcinoma (READ), with AUC values all exceeding 0.8. Additionally, *PLXNA3* showed exceptionally high AUC values for Cholangiocarcinoma (CHOL) across both TCGA and TCGA-GTEx datasets (AUC > 0.9), suggesting potential pan-digestive tract utility (**Figure 5B**).

Expression comparisons between tumor and normal tissues from TCGA confirmed significant upregulation of *PLXNA3* in gastrointestinal cancers. Wilcoxon test results showed consistently higher expression levels in COAD, READ, STAD, and ESCA tumors compared to matched normal tissues (p < 0.001 for all; **Figure 5C**). These findings were supported by transcriptomic data from the Human Protein Atlas, which reported markedly higher expression of *PLXNA3* in gastrointestinal tumors relative to normal counterparts—colorectal cancer (9.5), gastric cancer (9.2), and rectal cancer (8.7), compared to corresponding normal tissues (7.2, 6.8, and 2.1, respectively) (**Figure 5D**).

To visualize intra-group organ-level expression trends, we standardized TPM data from TCGA and GTEx using Z-score transformation and mapped median scores onto a human anatomical atlas via the gganatogram package. In normal tissues, *PLXNA3* expression was primarily enriched in the colorectal region of the gastrointestinal tract, whereas in tumor tissues, its expression was concentrated in gastric and esophageal organs (**Supplementary Figure 2**).

Finally, paired sample analyses within TCGA confirmed significantly higher *PLXNA3* expression in tumor tissues compared to adjacent non-tumor controls across COAD, READ, STAD, and ESCA. This pattern was particularly consistent in colorectal cancer (**Figure 5E**), reinforcing *PLXNA3*’s relevance as a tumor-associated gene within the digestive tract.

### 3.5 Prognostic survival analysis of *PLXNA3* in pan-gastrointestinal cancers

To further investigate the prognostic relevance of *PLXNA3*, we performed survival analyses across gastrointestinal malignancies using both univariate Cox regression and KM survival analyses. These analyses covered four major survival metrics: overall survival (OS), disease-specific survival (DSS), progression-free interval (PFI), and disease-free interval (DFI).

**Figure 6:**
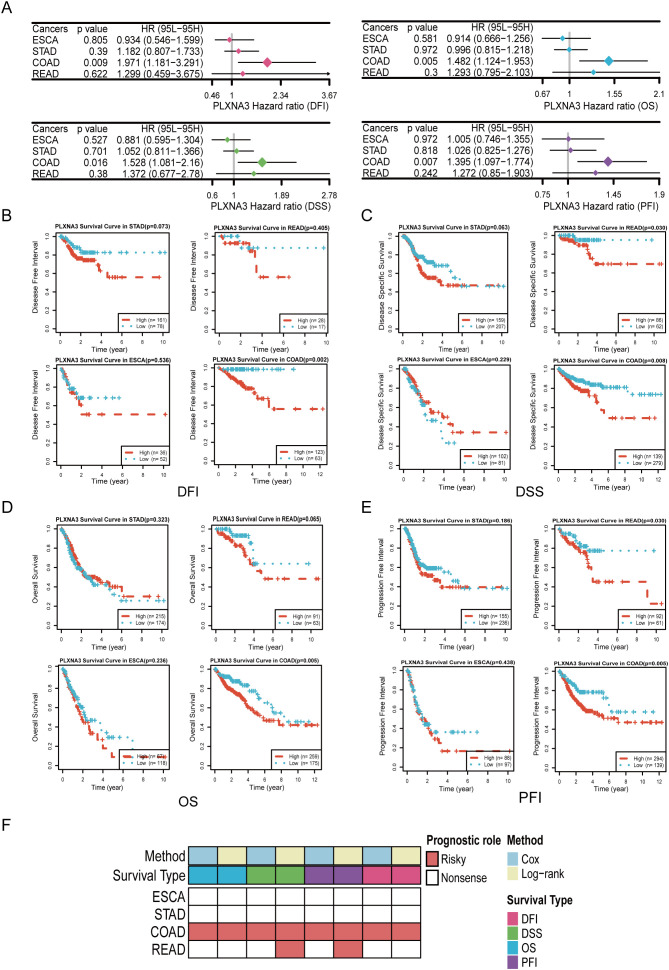
Survival analyses of *PLXNA3* across gastrointestinal malignancies. **(A)** Forest plot of univariate Cox regression analyses evaluating *PLXNA3* expression in relation to OS,DSS, PFI, and DFI across gastrointestinal cancers.**(B-E)** Kaplan–Meier survival curves showing that high *PLXNA3* expression is significantly associated with poorer DFI **(B)**, DSS **(C)**, OS **(D)**, and PFI **(E)** in COAD (p < 0.05).**(F)** Heatmap summarizing prognostic significance of *PLXNA3* across cancers and survival metrics. Red blocks indicate significant risk associations (HR > 1) validated by both Cox and KM methods, with COAD showing the most consistent adverse association.

In the univariate Cox model, *PLXNA3* consistently appeared as a high-risk factor (HR >1) across several cancer types, with particularly strong statistical significance in COAD (**Figure 6A**). KM analyses further confirmed this trend: high *PLXNA3* expression was significantly associated with poor prognosis across all survival endpoints in COAD (p < 0.05 for OS, DSS, PFI, and DFI; **Figures 6B–E**).

To consolidate these findings, we compiled a summary heatmap integrating Cox and log-rank results across multiple gastrointestinal tumors (**Figure 6F**). *PLXNA3* stood out as a robust and consistent risk gene in COAD across both analytical approaches, while READ demonstrated partial significance in specific endpoints.

### 3.6 Spatial transcriptomic analysis of *PLXNA3* in colorectal cancer

**Figure 7:**
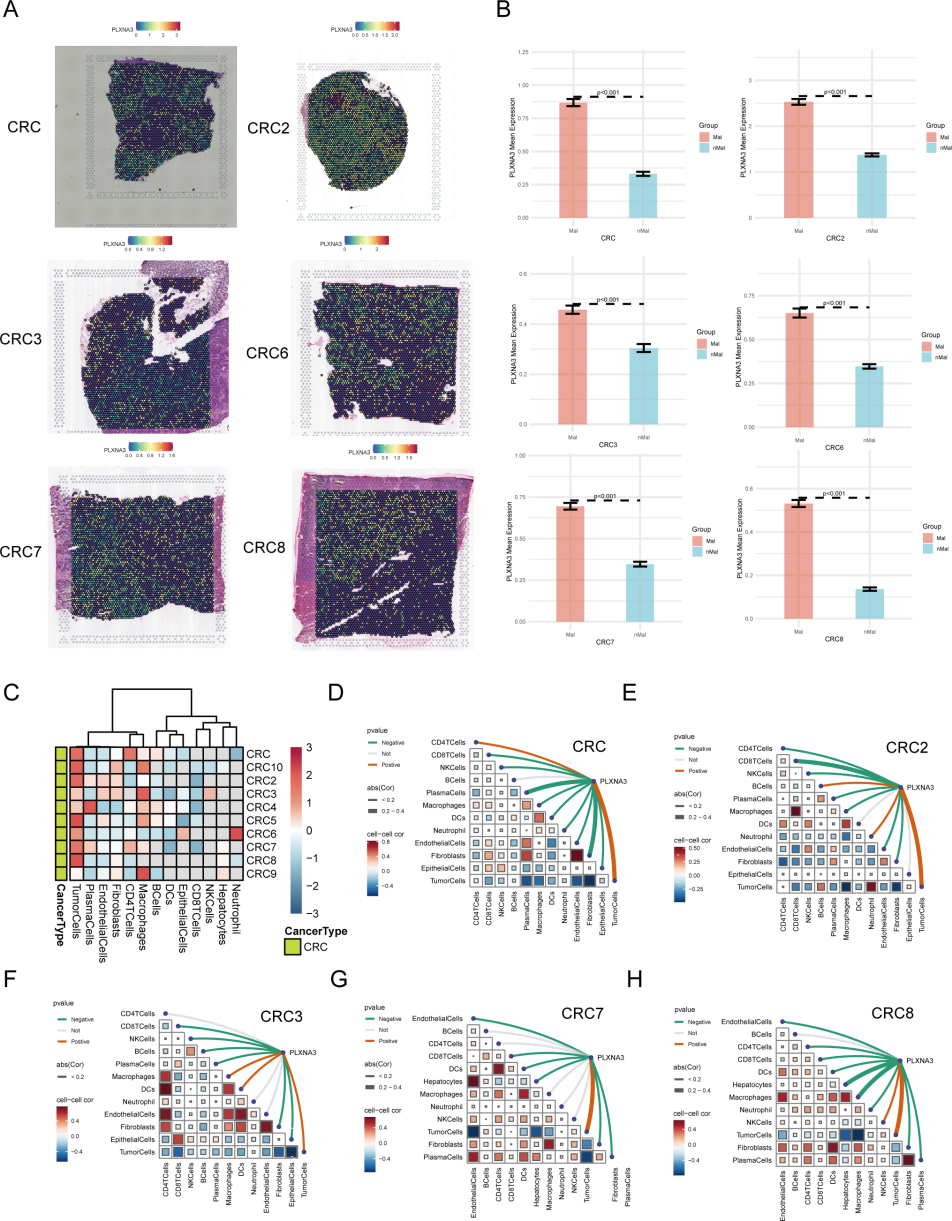
Spatial transcriptomic profiling of *PLXNA3* in colorectal cancer (CRC). **(A)** SpatialFeaturePlot visualizations showing *PLXNA3* expression across six primary CRC samples (CRC/CRC2/CRC3/CRC6/CRC7/CRC8). **(B)** Bar plots comparing *PLXNA3* expression between Mal and nMal regions. All samples show significantly higher expression in malignant zones (Wilcoxon test, p < 0.05). **(C)** Heatmap displaying *PLXNA3* expression across dominant cell-type zones (e.g., malignant cells, endothelial cells, immune cells). *PLXNA3* is primarily enriched in malignant regions and suppressed in immune-dense zones such as plasma cells and CD8^+^ T cells. **(D-H)** Spearman correlation matrix summarizing the relationship between *PLXNA3* expression and microregional cellular composition across 6 CRC spatial samples. *PLXNA3* shows strong positive correlations with malignant zones and negative correlations with immune-associated regions.

Given its prognostic significance in COAD and READ, we next explored the spatial expression patterns of *PLXNA3* in CRC using spatial transcriptomic data. Normalized expression data from ten CRC tissue sections were analyzed using Seurat’s SpatialFeaturePlot function. Spatial visualization revealed that *PLXNA3* was predominantly expressed in tumor-enriched regions across all primary CRC samples (**Figure 7A**; **Supplementary Figure 3A**).

To quantify this spatial localization, we divided each tissue section into Mal and nMal zones based on the proportion of malignant cells. In all CRC samples, *PLXNA3* expression was significantly higher in malignant regions compared to non-malignant areas (p < 0.05; **Figure 7B** and **Supplementary Figure 3B**), underscoring its spatial enrichment in tumor tissue.

Further, we analyzed *PLXNA3* expression across spatially defined cell-type dominant regions (**Figure 7C**). Heatmaps showed that *PLXNA3* was enriched in malignant cell regions, while expression in immune-associated regions (e.g. plasma cells, CD8^+^ T cells, CD4^+^ T cells) was consistently lower. This spatial distribution mirrors prior survival and immune correlation results, reinforcing its role in tumor-specific proliferation.

Finally, Spearman correlation analysis across all 10 samples (**Figures 7D-H** and **Supplementary Figure 3C**) revealed that *PLXNA3* expression was most positively correlated with malignant zones and negatively associated with immune-dense regions. Furthermore, negative correlation could be found between tumor cells and immune-associated cells such as CD8^+^ T cells, CD4^+^ T cells and plasma. These findings highlight the gene’s spatial specificity and support its potential role in promoting tumor progression and shaping immune exclusion in CRC.

### 3.7 Single-cell analysis of *PLXNA3* in colorectal and pan-cancer contexts

Following the spatial transcriptomic observation of *PLXNA3* enrichment in malignant epithelial regions, we performed a single-cell level analysis across gastrointestinal tumors to evaluate its expression in diverse cell types, particularly immune populations. Using the TISCH2 database, we selected three high-quality colorectal cancer datasets—GSE166555, EMTAB8107, and GSE146771—for integrated analysis.

**Figure 8:**
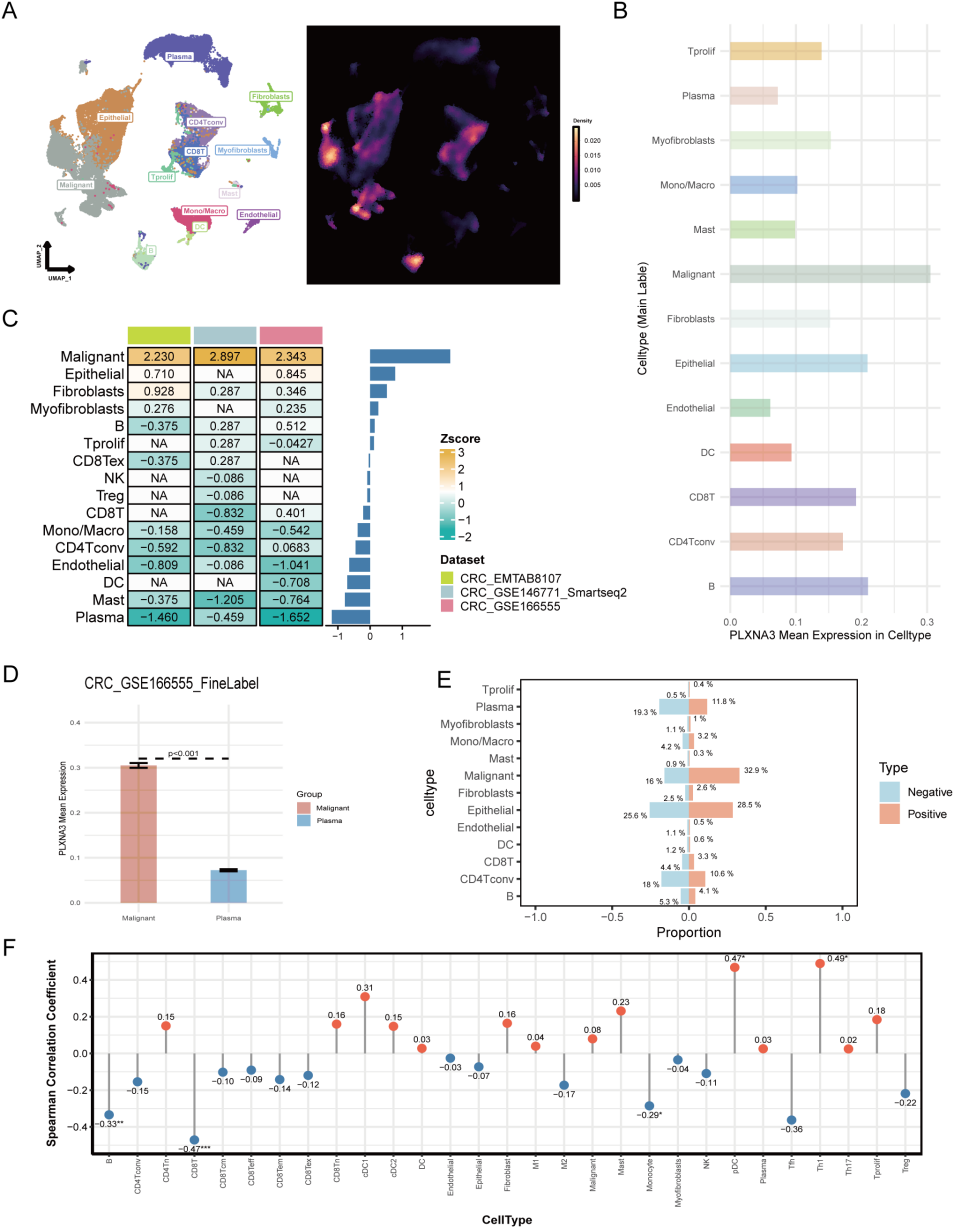
Single-cell landscape of *PLXNA3* expression in colorectal and pan-cancer contexts. **(A)** UMAP plot showing cell type clustering (left) and *PLXNA3* expression density (right) in CRC dataset GSE166555. **(B)** Mean expression of *PLXNA3* across cell types in GSE166555. **(C)** Heatmap of *PLXNA3* expression Z-scores in 13 cell types across three CRC datasets (GSE166555, EMTAB8107, and GSE146771). **(D)** Boxplot comparing *PLXNA3* expression between malignant cells and plasma cells in GSE166555 (Wilcoxon test, p < 0.001). **(E)** Proportional composition of major cell types in *PLXNA3* ^+^ vs. *PLXNA3* ^-^ groups. Immune cells are enriched in the *PLXNA3* ^-^ group, while malignant cells dominate the *PLXNA3* ^+^ group in GSE166555. **(F)** Spearman correlation coefficients between *PLXNA3* expression and relative abundance of immune/stromal cell types across all TISCH2 pan-cancer datasets. Positive correlations observed with pDCs and Th1 cells; negative correlations with CD8^+^ T cells, B cells, and monocytes.

UMAP-based clustering revealed epithelial and malignant cells as predominant populations (**Figure 8A** and **Supplementary Figure 4A**). *PLXNA3* was highly expressed in these cell types, with markedly lower expression in plasma cells and mast cells, as shown by the gene-level heatmap (**Figure 8A** and **Supplementary Figure 4A**). Bar plots further confirmed elevated *PLXNA3* expression in malignant and fibroblast cells, while immune subsets such as plasma cells, DC, and monocyte exhibited minimal expression (**Figure 8B** and **Supplementary Figure 4B**).

We next used Z-score transformation across all three datasets to assess the relationship between *PLXNA3* expressions and specific cell type abundances (**Figure 8C**). *PLXNA3* was consistently and positively correlated with malignant cells and negatively correlated with plasma cells. Boxplots comparing *PLXNA3* expression between malignant and plasma cells in all datasets revealed highly significant differences (p < 0.001; **Figure 8D** and **Supplementary Figure 4C**).

To further explore the distributional impact of *PLXNA3* expression, we stratified cells into *PLXNA3* ^+^ and *PLXNA3* ^-^ groups and compared their cellular composition. In the *PLXNA3*-negative group, immune cells—including CD4^+^ T cells, monocytes, and plasma cells—were significantly enriched, while malignant cell proportions were markedly reduced (**Figure 8E**).

Finally, to validate these findings on a broader scale, we examined all single-cell datasets within TISCH2. Lollipop plots of Spearman correlation coefficients between *PLXNA3* expression and cell type fractions across datasets showed consistent trends: *PLXNA3* expression positively correlated with pDCs and Th1 cells, but negatively correlated with monocytes, B cells, and CD8^+^ T cells (**Figure 8F**).

### 3.8 Multi-layer regulatory and functional profiling of *PLXNA3* in gastrointestinal cancers

To further elucidate the mechanistic basis by which *PLXNA3* may contribute to tumor progression and immune suppression, we performed a multi-layered regulatory and functional analysis across gastrointestinal cancers.

**Figure 9:**
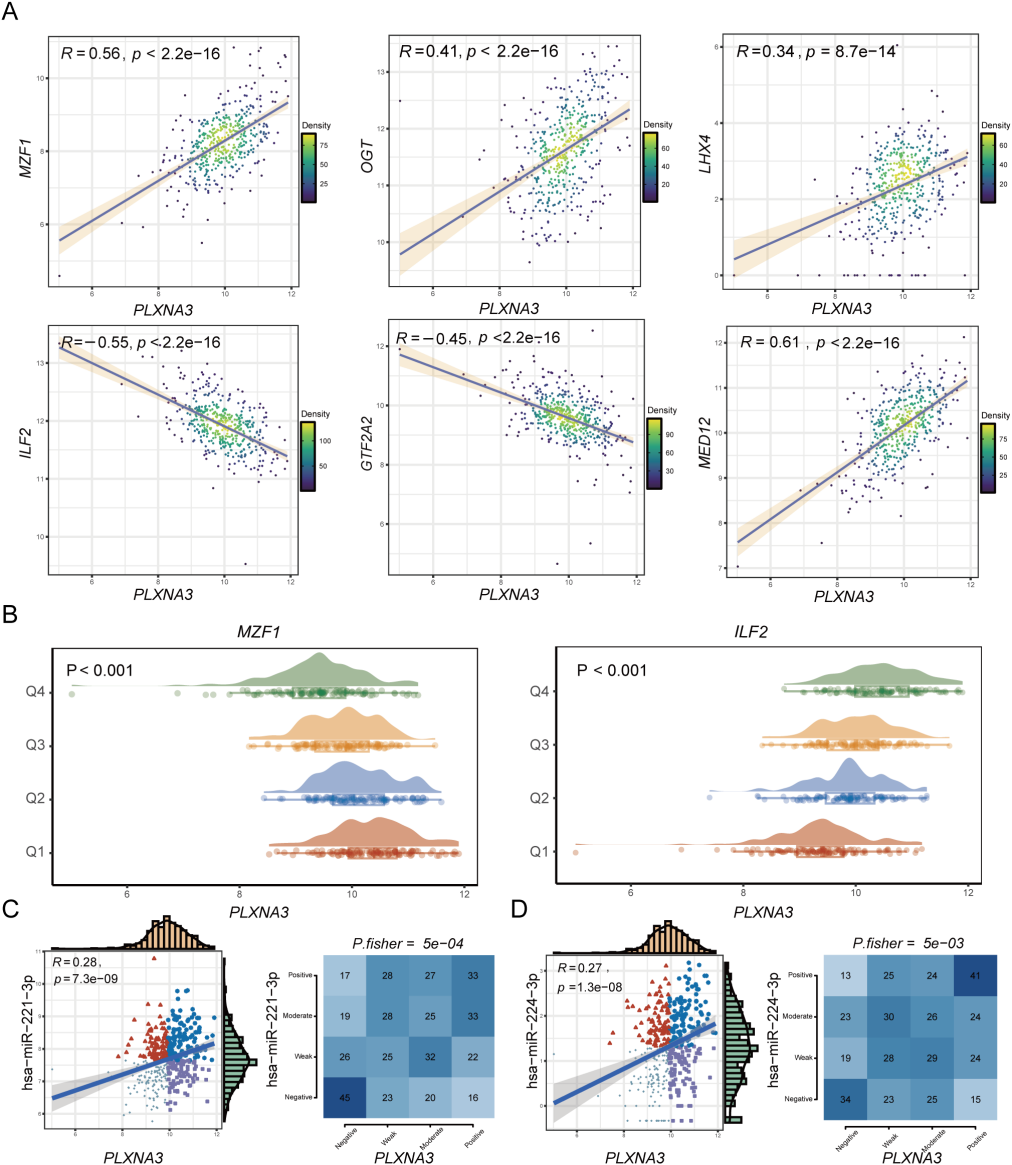
Transcriptional and post-transcriptional regulatory analysis of *PLXNA3* in colorectal cancer**. (A)** Pearson correlation analysis between *PLXNA3* and selected TFs in COAD, showing strong positive correlations with MZF1, OGT, LHX4, MED12, and negative correlations with ILF2 and GTF2A2. **(B)** Expression distributions of *PLXNA3* stratified by TF expression quartiles (Q1–Q4), using MZF1 and ILF2 as examples. **(C, D)** Correlation between *PLXNA3* and two oncogenic miRNAs—*hsa-miR-221-3p* **(C)** and *hsa-miR-224-3p* **(D)**—including Pearson correlation coefficients, scatter plots, and Fisher’s exact test results for categorical expression levels.

We first examined transcriptional regulation by identifying the top 25 transcription factors (TFs) most positively and negatively correlated with *PLXNA3* expression in each TCGA cancer type (**Supplementary Figure 5**). In COAD, Pearson correlation analysis revealed strong positive correlations between *PLXNA3* and MZF1, OGT, LHX4, and MED12, while ILF2 and GTF2A2 showed strong negative correlations (**Figure 9A**). Stratification analysis based on quartile expression levels (Q1–Q4) of the selected TFs further confirmed the relationship with *PLXNA3* expression, demonstrating consistent patterns with the correlation results (**Figure 9B**).

We next explored post-transcriptional regulation of *PLXNA3* using miRNA correlation data from the grswsci platform (https://grswsci.top/). Among the predicted miRNAs, hsa-miR-221-3p and hsa-miR-224-3p exhibited notable positive associations with *PLXNA3* expression. These findings were validated through Pearson correlation and Fisher’s exact test, both showing significant associations (**Figures 9C, D** and **Supplementary Table 1**).

**Figure 10:**
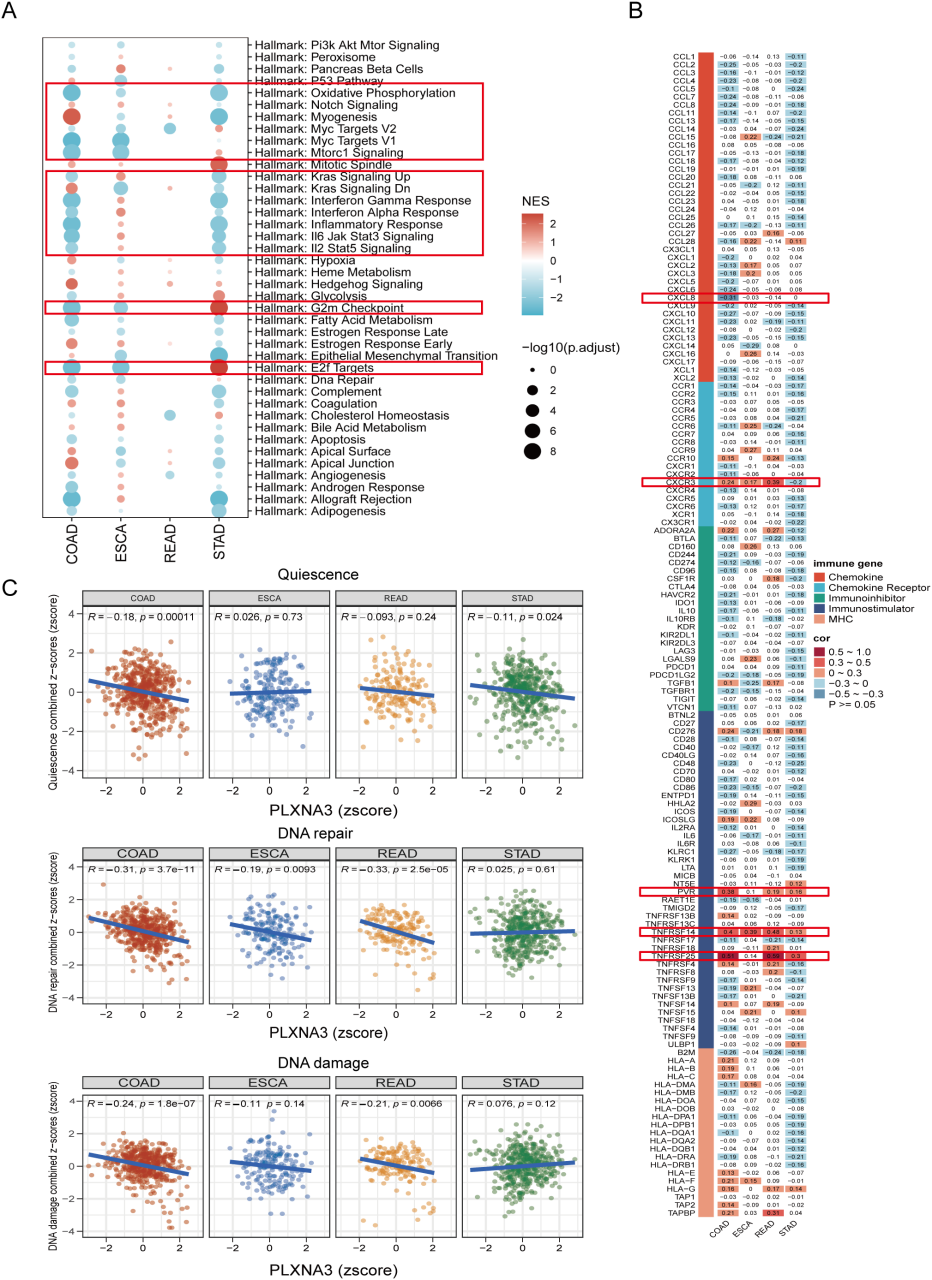
Functional pathway and immune gene correlation analysis of *PLXNA3* in gastrointestinal cancers. **(A)** GSEA bubble plot showing *PLXNA3*-associated Hallmark pathways across COAD, ESCA, READ, and STAD, with significant enrichment in inflammatory, interferon, Myc target, oxidative phosphorylation, and E2F-related pathways.**(B)** Heatmap of Pearson correlation between *PLXNA3* and immune-related genes across four gastrointestinal cancer types, highlighting associations with *CXCL8, CXCR3, PVR, TNFRSF14*, and *TNFRSF25*.**(C)** Correlation between *PLXNA3* and tumor functional states—quiescence, DNA repair, and DNA damage—based on CancerSEA gene set scoring across COAD, ESCA, READ, and STAD.

To identify functional pathways potentially modulated by *PLXNA3*, we performed GSEA using both Hallmark and KEGG gene sets across COAD, ESCA, READ, and STAD (**Figure 10A** and **Supplementary Figure 6**). In COAD, *PLXNA3* expression was negatively associated with key oncogenic and immunoregulatory pathways including Interferon Gamma Response, KRAS signaling, Oxidative Phosphorylation, Myc Targets V1, Inflammatory Response, and E2F Targets.

We then evaluated co-expression patterns between *PLXNA3* and immune-related genes using Pearson correlation analysis and visualized the results via heatmap (**Figure 10B**). Notably, *PLXNA3* was significantly correlated with several key immune modulators in COAD, including CXCL8, CXCR3, PVR, TNFRSF14, and TNFRSF25, indicating its potential role in modulating immune infiltration and immune checkpoint signaling.

Lastly, to assess how *PLXNA3* links with broader tumor phenotypes, we integrated CancerSEA functional state scores and conducted correlation analysis across 14 tumor-related biological processes (**Supplementary Figure 7**). Focused analysis in gastrointestinal cancers revealed that in both COAD and READ, *PLXNA3* exhibited strong negative correlations with DNA repair, and DNA damage pathways, while in COAD, *PLXNA3* exhibited certain negative correlations with quiescence (**Figure 10C**), further supporting its functional role in tumor aggressiveness.

### 3.9 Immunohistochemical validation and drug sensitivity analysis of *PLXNA3* in CRC

**Figure 11:**
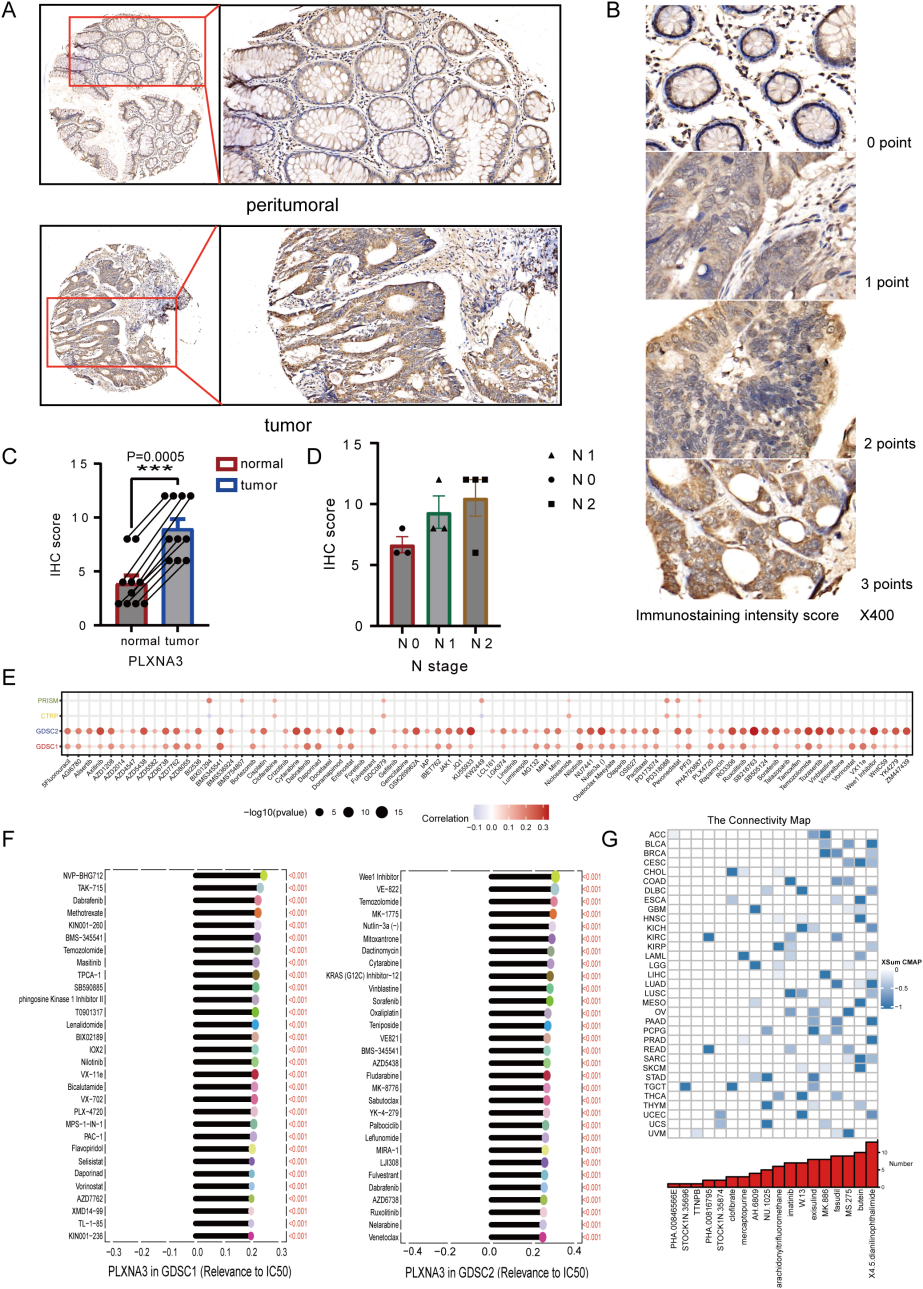
Immunohistochemical validation and drug sensitivity analysis of *PLXNA3* in colorectal cancer. **(A)** Representative IHC staining of *PLXNA3* in paired tumor and adjacent normal CRC tissues (magnifications: 5×, 20×). **(B)** Reference panel of staining intensity scores (0–3) used for IHC scoring. **(C)** Boxplot comparing IHC scores between tumor and normal tissues. *PLXNA3* expression is significantly higher in tumor tissues (p = 0.0005). **(D)** Scatter plots showing IHC scores in relation to tumor TNM stage. A positive trend is observed between lymph node (N) stage and *PLXNA3* levels. **(E)** Correlation heatmap between *PLXNA3* expression and drug response across four sensitivity datasets (PRISM, CTRP, GDSC1, GDSC2), with strongest associations in GDSC1 and GDSC2. **(F)** Identification of individual compounds showing significant positive correlations between *PLXNA3* expression and sensitivity to specific chemotherapeutics. **(G)** CMap analysis revealing candidate small molecules with potential to reverse *PLXNA3*-driven transcriptional programs. Compounds with the lowest connectivity scores are highlighted. The asterisks (*, **, ***) in the figures indicate statistical significance levels: p < 0.05 (*), p < 0.01. (**), p < 0.001 (***).

To validate our bioinformatics findings at the protein level, we performed IHC staining for *PLXNA3* in 22 clinical samples of colorectal cancer. Representative images demonstrated markedly increased staining intensity in tumor tissues compared to paired peritumoral tissues, both at 5× and 20× magnifications (**Figure 11A**). Staining intensity was graded into four levels (0–3 points), as shown in high-magnification examples (**Figure 11B**). IHC scores were calculated as the product of staining intensity and percentage of positive cells. Quantitative comparison between tumor and adjacent normal tissues revealed significantly higher *PLXNA3* IHC scores in tumor samples (p = 0.0005; **Figure 11C**), supporting the translational relevance of our transcriptomic predictions.

To preliminarily explore potential clinical relevance, we plotted IHC scores against TNM staging parameters. Although sample size precluded statistical testing, a trend of increasing *PLXNA3* expression with advancing N stage was observed (**Figure 11D**), suggesting possible association with nodal metastasis and tumor progression.

To investigate the potential druggability of *PLXNA3*, we assessed its correlation with compound sensitivity across multiple pharmacogenomic databases. Among PRISM, CTRP, GDSC1, and GDSC2 datasets, the strongest correlations were observed in GDSC1 and GDSC2, where *PLXNA3* expression exhibited significant positive associations with drug half maximal inhibitory concentration (IC50) values (**Figure 11E**), indicating reduced drug sensitivity in *PLXNA3*-high contexts. Subsequent analysis identified several compounds whose IC50 values were positively correlated with *PLXNA3* expression in both datasets, including mTOR and PI3K pathway inhibitors (**Figure 11F**).

Finally, to identify compounds that might reverse *PLXNA3*-associated transcriptional programs, we performed a CMap analysis using the XSum algorithm. A total of 1288 small molecules were scored based on their inverse similarity to *PLXNA3*-related gene signatures. Among these, compounds with the most negative scores—suggestive of therapeutic potential—were enriched in kinase inhibitors and differentiation inducers (**Figure 11G**).

## 4 Discussion

In this study, we developed a robust immune-related prognostic model for CRC by integrating transcriptomic and clinical data from TCGA and GEO cohorts using over 100 machine learning algorithms. This ensemble-based model achieved strong predictive performance and highlighted *PLXNA3* as a top-ranking risk gene with consistent prognostic significance across gastrointestinal cancers. Multi-level validations—spanning spatial transcriptomics, single-cell analysis, and TF expression—confirmed its elevated expression in tumor tissues and its enrichment in malignant epithelial compartments.

*PLXNA3*, a member of the plexin family originally implicated in axon guidance (9), has recently been linked to oncogenic processes in breast cancer and nephroblastoma (11, 12). Consistent with prior studies suggesting its upregulation correlates with poor prognosis in various cancers (10), our analysis extended its significance to gastrointestinal malignancies, particularly COAD. Notably, spatial and single-cell analyses revealed a mutually exclusive distribution pattern between *PLXNA3* and immune-rich regions, especially CD8^+^ T cells, suggesting a role in immune evasion. Furthermore, we identified a strong negative correlation between *PLXNA3* and plasma cells in CRC, although this finding requires further validation in larger cohorts.

Our transcription factor analysis uncovered several TFs highly correlated with *PLXNA3* expression in COAD. Among them, MZF1, OGT, LHX4, and MED12 have been implicated in tumor stemness, drug resistance, and immune escape mechanisms (38–41). In contrast, GTF2A2 and ILF2—downregulated in *PLXNA3*-high tumors—play roles in transcriptional activation and T cell signaling (42, 43), respectively, suggesting a potential immunosuppressive reprogramming in *PLXNA3*-high tumors. Additionally, two well-established oncogenic miRNAs, hsa-miR-221-3p and hsa-miR-224-3p, were positively correlated with *PLXNA3* expression. Given their roles in shaping the tumor immune microenvironment (44–46), their association with *PLXNA3* supports its relevance in immune modulation. Furthermore, hsa-miR-224-3p was found upregulated in serum and tissues of colorectal cancer patients with lymph node metastasis, suggesting that it could be used as a marker to predict progression (47).

Functionally, GSEA revealed that *PLXNA3* expression in COAD is negatively associated with pathways such as Interferon Gamma Response, Oxidative Phosphorylation, and Inflammatory Response, which are central to T cell activation and recruitment (48–50). Moreover, *PLXNA3* expression was negatively correlated with KRAS signaling UP and positively correlated with KRAS signaling DN gene sets, suggesting a potential suppression of classical Ras pathway activity. This is consistent with structural evidence that *PLXNA3* possesses Ras-GAP activity, particularly toward R-Ras and M-Ras, which leads to the attenuation of downstream RAS–RAF–MAPK signaling (51). These results suggest that *PLXNA3* may suppress classical RAS signaling activity and influence tumor progression through non-canonical pathways beyond KRAS activation. As a gene closely related to CRC, the relationship between *RAF* and *PLXNA3* remains to be further explored; however, evidence from *PLXNA1* suggests a potential link between plexins and RAF-mediated signaling (52). In addition, while *PLXNA3* showed a weak negative correlation with most immune-related genes, it was positively associated with *PVR*, *TNFRSF14*, and *TNFRSF25*, which are known to foster immunosuppressive niches (53–57). In tumor cells, *PLXNA3* showed consistent negative correlations with pathways that are typically associated with tumor-suppressive functions, including DNA repair, DNA damage response, and quiescence (58, 59). By concurrently modulating oncogenic signaling and impairing anti-tumor immunity via CD8^+^ T cell dysfunction and immune exclusion, along with suppressing tumor-protective cellular program, *PLXNA3* may play a dual role in driving colorectal cancer progression both through immune evasion and intrinsic cellular aggressiveness.

The IHC results further validated *PLXNA3* overexpression at the protein level, and a trend of increasing expression with higher N stages suggested a potential link to lymph node metastasis. Drug sensitivity profiling across GDSC1 and GDSC2 databases revealed widespread positive correlations between *PLXNA3* expression and drug IC50 values, indicating a potential role in therapeutic resistance. Finally, using CMap analysis, we identified several candidate compounds—including imatinib, fasudil, and MS-275—that may reverse *PLXNA3*-driven transcriptional programs. While the exact mechanisms remain to be elucidated, MS-275’s inhibition of HDAC1/3 (60) and known effects on IFN-γ signaling (61), antigen presentation, and T cell recruitment (62) lend biological plausibility to its potential efficacy against *PLXNA3*-high tumors. Meanwhile, imatinib exerts crucial immunomodulatory functions. It suppresses IDO, leading to enhanced intertumoral CD8^+^ T cell activation and Treg apoptosis, promotes DC maturation and CD8^+^ T cell infiltration, and inhibits M2 macrophage polarization (63–65).As for fasudil, remodels the tumor immune microenvironment by multiple mechanisms: it enhances phagocytosis by antigen-presenting cells and promotes dendritic cell maturation, leading to potent CD8^+^ T cell priming and infiltration (66). These findings also indirectly support our previous discovery of a potential association between *PLXNA3* and T cells.

Despite these strengths, our study has limitations. The reliance on public datasets may introduce batch effects and clinical heterogeneity. Moreover, functional validation *in vivo* and prospective clinical cohorts is essential to establish *PLXNA3* as a reliable therapeutic target. Notably, we did not directly assess the predictive value of *PLXNA3* in the context of immune checkpoint inhibitor (ICI) therapy, due to the lack of large-scale, colorectal cancer–specific immunotherapy cohorts with available treatment outcome data. While many recent studies have used immunotherapy cohorts from other cancer types for CRC-related analyses, such extrapolations are inherently limited (67, 68). Moreover, current immunotherapy decision-making in colorectal cancer is primarily based on MSI-H/dMMR status rather than gene-level expression (69). Nonetheless, our findings provide a meaningful background for future investigations, and we plan to validate the role of *PLXNA3* in immunotherapy through well-designed *in vitro* and *in vivo* experiments. In conclusion, the integration of multi-omics analyses—encompassing survival modeling, spatial and single-cell transcriptomics, immunohistochemistry, and pharmacogenomic screening—positions *PLXNA3* as a promising biomarker for prognosis and immunotherapy response in colorectal cancer.

## Data Availability

All datasets analyzed in this study are publicly available unless otherwise noted. Transcriptomic and clinical data were retrieved from TCGA (https://portal.gdc.cancer.gov/) and GEO (https://www.ncbi.nlm.nih.gov/geo/), with the GEO dataset GSE39582 used for model construction. Normal tissue transcriptome data were obtained from the GTEx (https://gtexportal.org/). Single-cell transcriptomic data were obtained from EMTAB8107, GSE108989, GSE112865, GSE120909, GSE122969, GSE136394, GSE139555, GSE146771, GSE166555, and GSE179784. Gene dependency and expression data were accessed via the DepMap portal (https://depmap.org/portal/). Protein and tissue-level expression data were sourced from the HPA (https://www.proteinatlas.org/). Spatial transcriptomic and immune cell composition data were obtained and analyzed using the Sparkle platform (https://grswsci.top/). Drug sensitivity data were derived from the GDSC1/2, PRISM, and CTRP datasets, with pharmacogenomic analysis also conducted via Sparkle. The IHC data generated from 22 colorectal cancer tissue samples collected at Fuzhou University Affiliated Provincial Hospital are not publicly available, but the corresponding IHC staining slide files can be obtained from the corresponding author upon reasonable request. No new sequencing data was generated in this study.

## Supplementary material

Supplementary Figure 1 Immune subtyping analysis showed no statistically significant distribution differences between groups

Supplementary Figure 2 Organ-level expression pattern of *PLXNA3* in normal and tumor tissues. Z-score normalized TPM values from GTEx and TCGA were visualized using the gganatogram package. *PLXNA3* expression was higher in the colorectal region among normal tissues and enriched in gastric and esophageal regions among tumor tissues.

Supplementary Figure 3 Spatial transcriptomic profiling of *PLXNA3* in colorectal cancer (CRC). **(A)** SpatialFeaturePlot visualizations showing *PLXNA3* expression across six primary CRC samples (CRC4/CRC5/CRC9/CRC10). **(B)** Violin plots comparing *PLXNA3* expression between malignant (Mal) and non-malignant (nMal) regions. **(C)** Spearman correlation matrix summarizing the relationship between *PLXNA3* expression and microregional cellular composition across rest 4 CRC spatial samples.

Supplementary Figure 4 Single-Cell Analysis of *PLXNA3* in Colorectal and Pan-Cancer Contexts. **(A)** UMAP plot showing cell type clustering (left) and *PLXNA3* expression density (right) in CRC dataset EMTAB8107 and GSE146771. **(B)** Mean expression of *PLXNA3* across cell types in EMTAB8107 and GSE146771. **(C)** Boxplot comparing *PLXNA3* expression between malignant cells and plasma cells in EMTAB8107 and GSE146771 (Wilcoxon test, p < 0.001). **(D)**Proportional composition of major cell types in *PLXNA3* ^+^ vs. *PLXNA3* ^-^ groups. Immune cells are enriched in the *PLXNA3* ^-^ group, while malignant cells dominate the *PLXNA3* ^+^ group in EMTAB8107 and GSE146771.

Supplementary Figure 5 top 25 TFs most positively and negatively correlated with *PLXNA3* expression in each TCGA cancer type

Supplementary Figure 6 GSEA bubble plot showing *PLXNA3*-associated KEGG and Hallmark pathways across COAD, ESCA, READ, and STAD, with significant enrichment in inflammatory, interferon, Myc target, oxidative phosphorylation, and E2F-related pathways.

Supplementary Figure 7 Correlation matrix shows *PLXNA3* associations with 14 tumor cell functional states from the CancerSEA database.

Supplementary Table 1 post-transcriptional regulation of *PLXNA3* using miRNA correlation data from the grswsci platform (https://grswsci.top/)

Supplementary Table 2 List of genes used for risk score calculation in the optimal machine learning model. This table summarizes the 17 genes selected by the highest-performing prognostic model (CoxBoost + SuperPC) and used to compute risk scores in both the training and validation cohorts.
