# Supplementary figures and images for: A machine learning-derived immune-related prognostic model identifies PLXNA3 as a functional risk gene in colorectal cancer
Source: Front Immunol. 2025 Sep 2;16:1653794. doi: 10.3389/fimmu.2025.1653794 (PMC12436408; doi:10.3389/fimmu.2025.1653794)

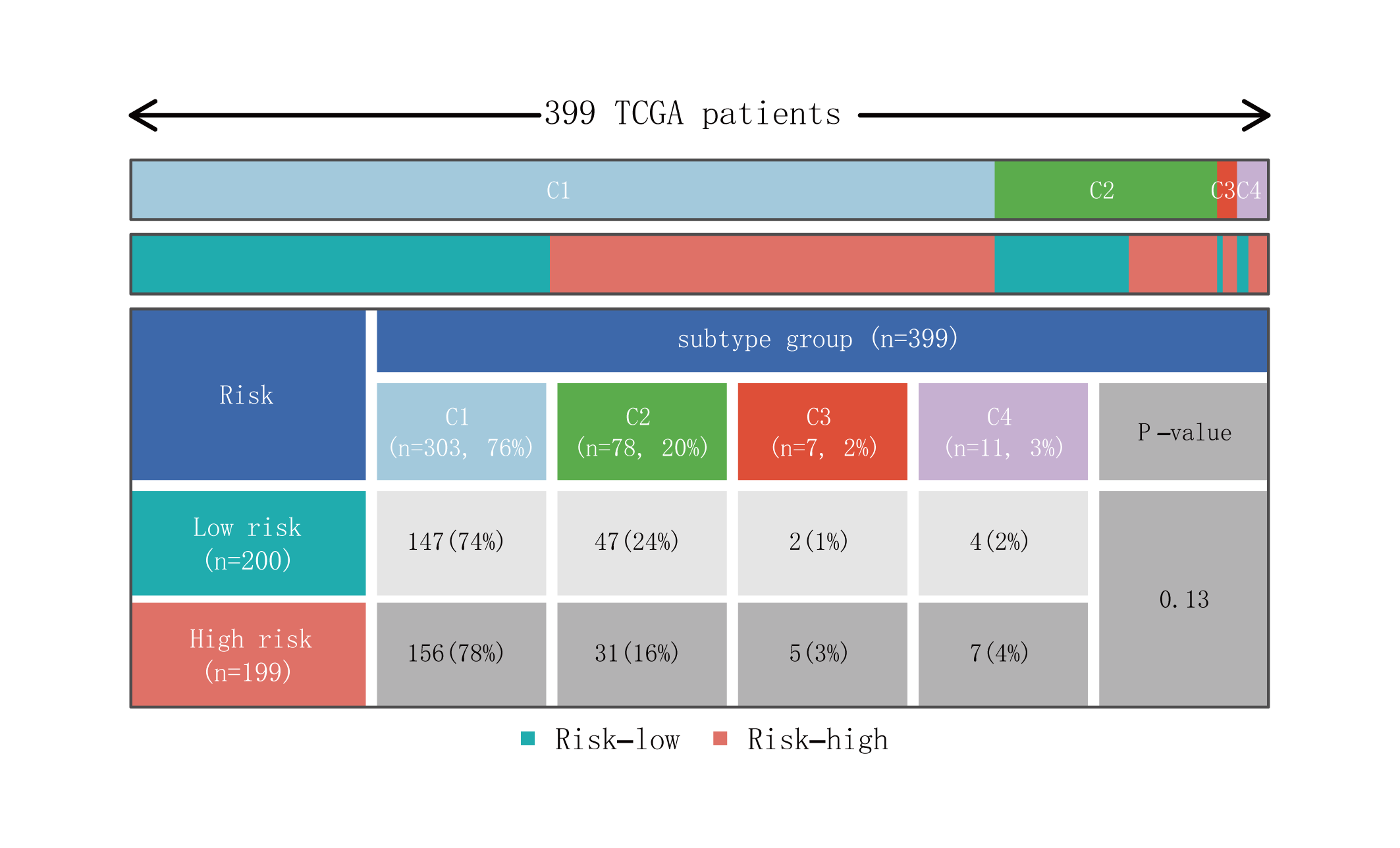

Supplement: Supplementary Figure 1 — Immune subtyping analysis showed no statistically significant distribution differences between groups [file Image1.tif]

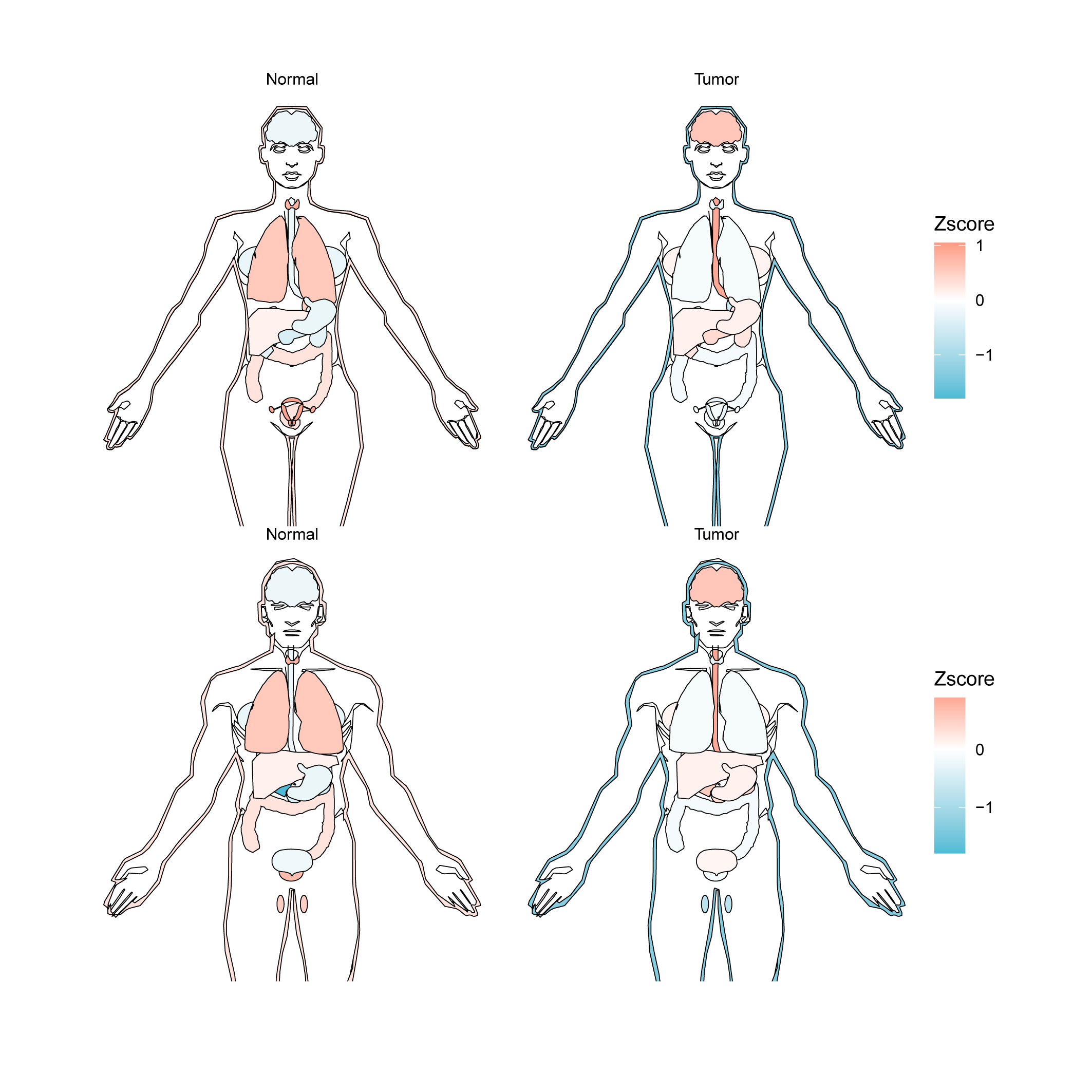

Supplement: Supplementary Figure 2 — Organ-level expression pattern of PLXNA3 in normal and tumor tissues. Z-score normalized TPM values from GTEx and TCGA were visualized using the gganatogram package. PLXNA3 expression was higher in the colorectal region among normal tissues and enriched in gastric and esophageal regions among tumor tissues. [file Image2.tif]

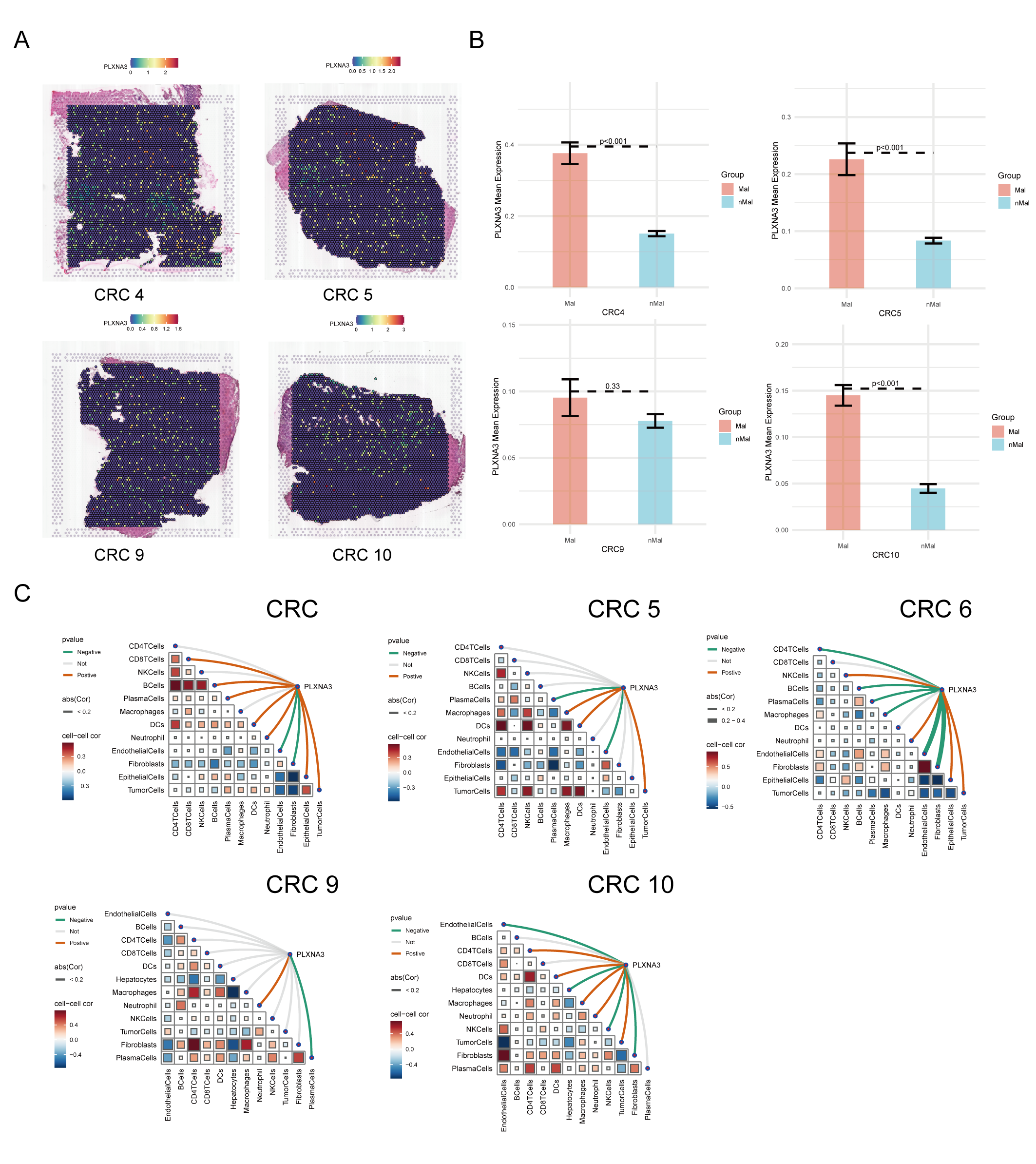

Supplement: Supplementary Figure 3 — Spatial transcriptomic profiling of PLXNA3 in colorectal cancer (CRC). (A) SpatialFeaturePlot visualizations showing PLXNA3 expression across six primary CRC samples (CRC4/CRC5/CRC9/CRC10). (B) Violin plots comparing PLXNA3 expression between malignant (Mal) and non-malignant (nMal) regions. (C) Spearman correlation matrix summarizing the relationship between PLXNA3 expression and microregional cellular composition across rest 4 CRC spatial samples. [file Image3.tif]

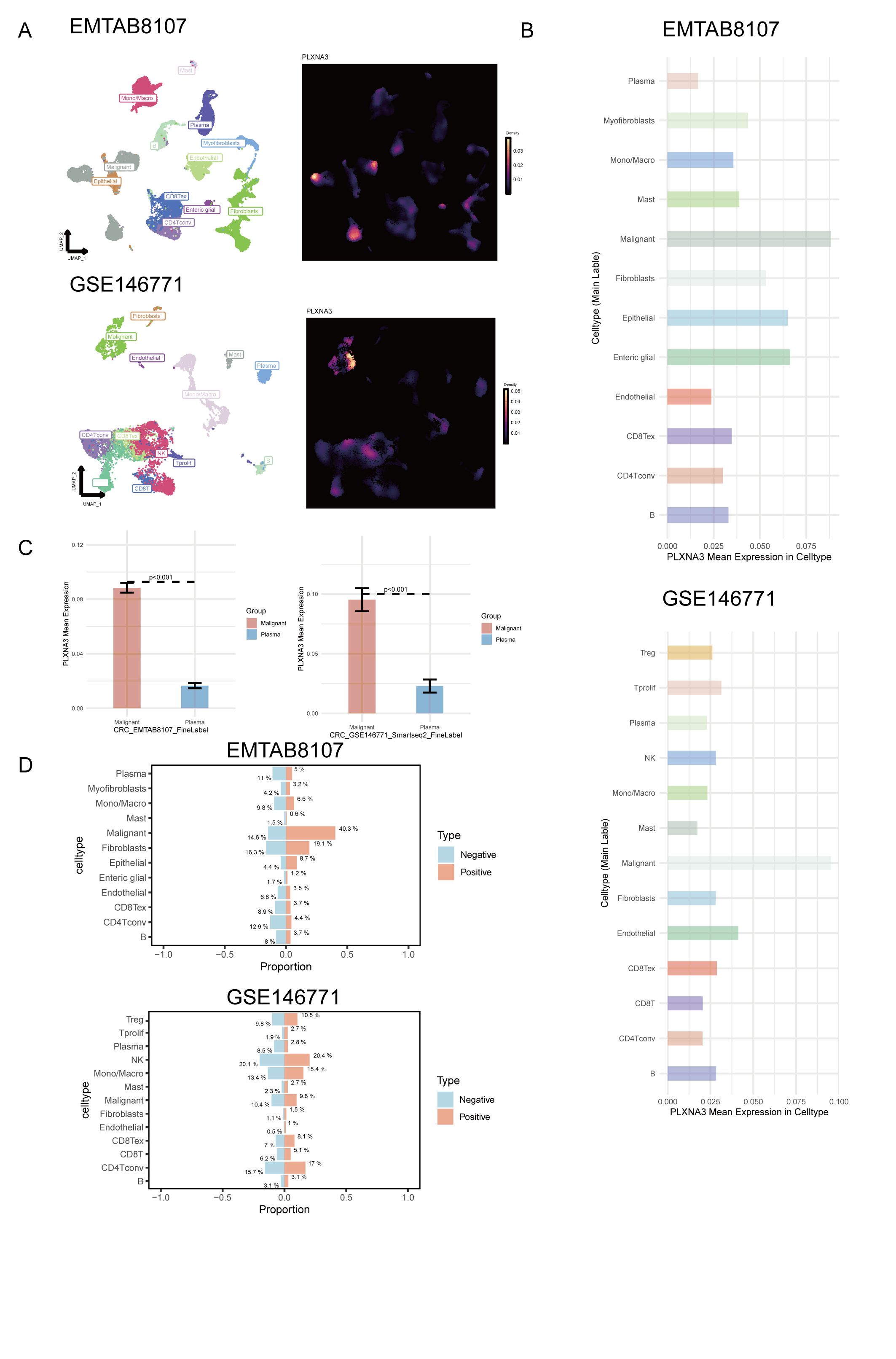

Supplement: Supplementary Figure 4 — Single-Cell Analysis of PLXNA3 in Colorectal and Pan-Cancer Contexts. (A) UMAP plot showing cell type clustering (left) and PLXNA3 expression density (right) in CRC dataset EMTAB8107 and GSE146771. (B) Mean expression of PLXNA3 across cell types in EMTAB8107 and GSE146771. (C) Boxplot comparing PLXNA3 expression between malignant cells and plasma cells in EMTAB8107 and GSE146771 (Wilcoxon test, p < 0.001). (D)Proportional composition of major cell types in PLXNA3 + vs. PLXNA3 - groups. Immune cells are enriched in the PLXNA3 - group, while malignant cells dominate the PLXNA3 + group in EMTAB8107 and GSE146771. [file Image4.tif]

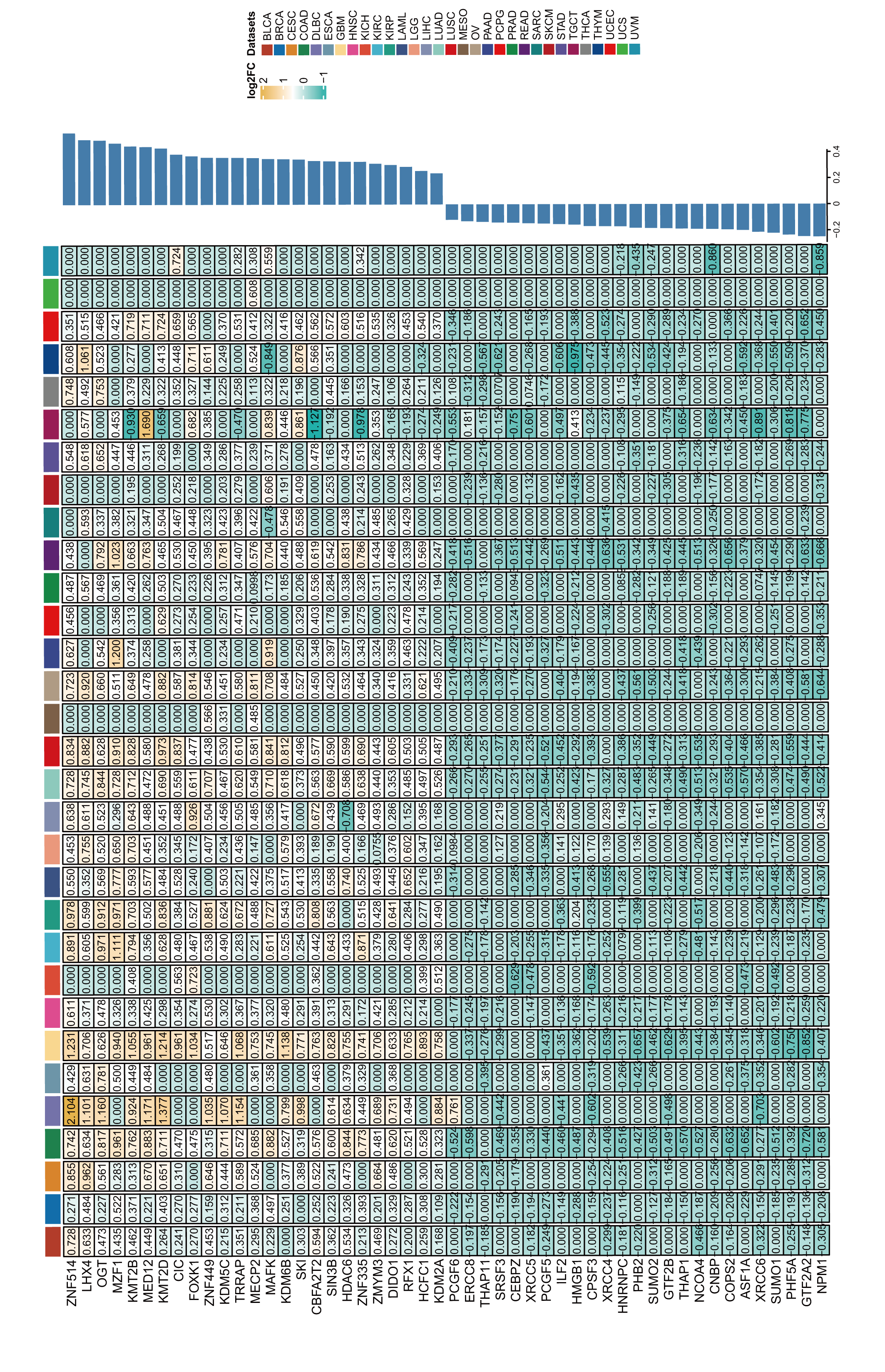

Supplement: Supplementary Figure 5 — top 25 TFs most positively and negatively correlated with PLXNA3 expression in each TCGA cancer type [file Image5.tif]

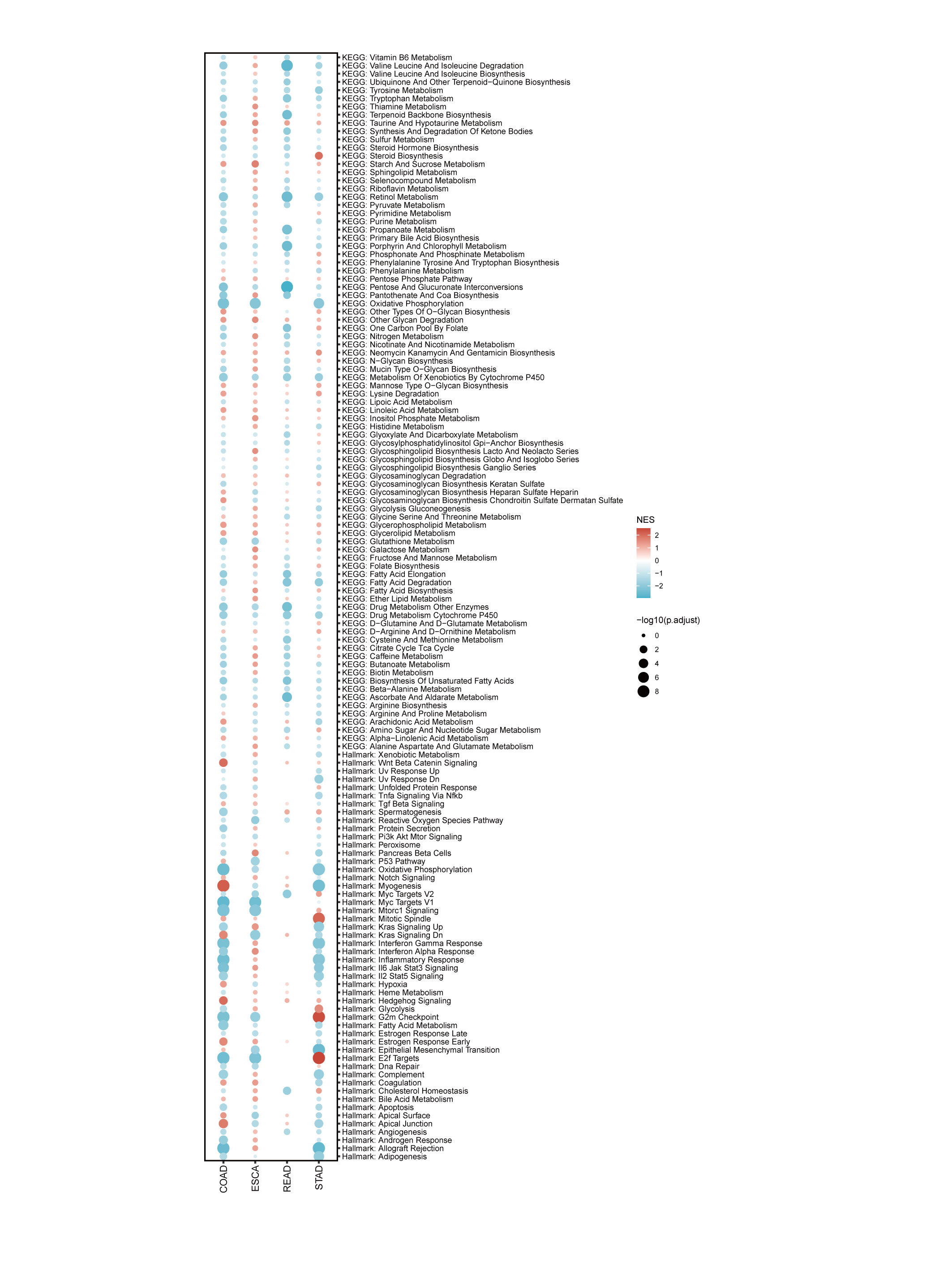

Supplement: Supplementary Figure 6 — GSEA bubble plot showing PLXNA3-associated KEGG and Hallmark pathways across COAD, ESCA, READ, and STAD, with significant enrichment in inflammatory, interferon, Myc target, oxidative phosphorylation, and E2F-related pathways. [file Image6.tif]

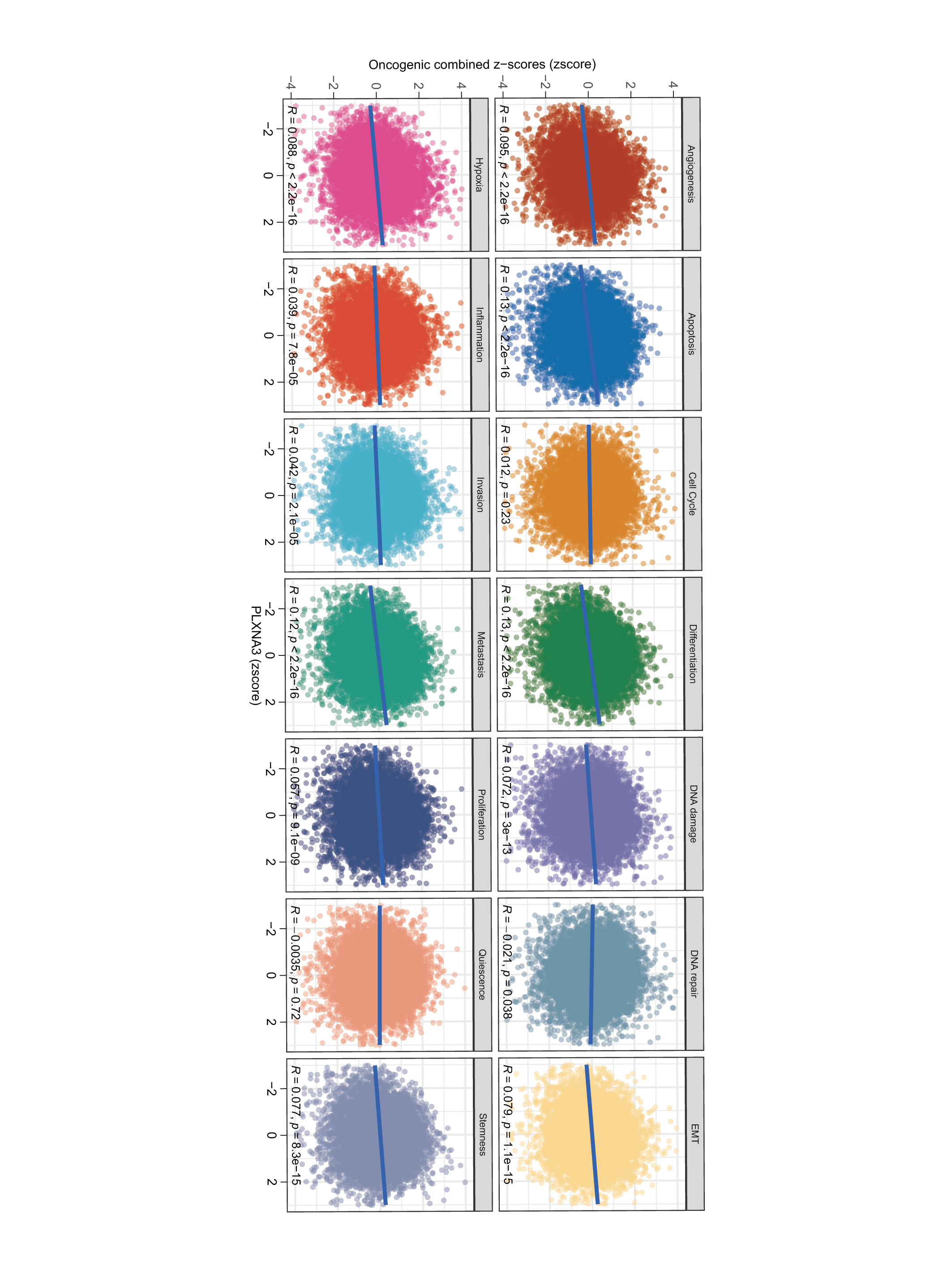

Supplement: Supplementary Figure 7 — Correlation matrix shows PLXNA3 associations with 14 tumor cell functional states from the CancerSEA database. [file Image7.tif]
